# Design, synthesis and computational study of new benzofuran hybrids as dual PI3K/VEGFR2 inhibitors targeting cancer

**DOI:** 10.1038/s41598-022-21277-2

**Published:** 2022-10-12

**Authors:** Omar A. El-Khouly, Morkos A. Henen, Magda A.-A. El-Sayed, Shahenda M. El-Messery

**Affiliations:** 1 Department of Pharmaceutical Organic Chemistry, Faculty of Pharmacy, Mansoura University, P.O. Box 35516, Mansoura, Egypt; 2 Faculty of Pharmacy, New Mansoura University, P.O. Box 35712, New Mansoura, Egypt; 3 Department of Biochemistry and Molecular Genetics, University of Colorado, Denver, USA; 4 Department of Pharmaceutical Chemistry, Faculty of Pharmacy, Horus University, P.O. Box 34518, New Damietta, Egypt

**Keywords:** Biological techniques, Cancer, Computational biology and bioinformatics, Drug discovery, Structural biology, Diseases, Oncology, Chemistry

## Abstract

Design and synthesis of a new series of benzofuran derivatives has been performed. ^1^H-NMR, ^13^C-NMR, elemental analysis, and IR were used to confirm the structures of the produced compounds. Hepatocellular carcinoma (HePG2), mammary gland breast cancer (MCF-7), epithelioid carcinoma cervical cancer (Hela), and human prostate cancer are used to test anticancer activity (PC3). In compared to DOX (4.17–8.87 µM), Compound **8** demonstrated the highest activity against HePG and PC3 cell lines, with an IC_50_ range of 11–17 µM. Compound **8** inhibited PI3K and VEGFR-2 with IC_50_ values of 2.21 and 68 nM, respectively, compared to 6.18 nM for compound LY294002 and 31.2 nM for compound sorafenib as PI3K and VEGFR-2 reference inhibitors, selectively. The molecular docking and binding affinity of the generated compounds were estimated and studied computationally utilizing molecular operating environment software as a PI3K and VEGFR-2 inhibitor (MOE). In conclusion, compound **8** exhibited significant action against hepatocellular and cervical cancer cell lines. Mechanistic study showed that it had a dual inhibitory effect against PI3K and VEGFR-2.

## Introduction

Cancer is firstly reported in Ancient Egypt in the Ebers papyrus which is an Egyptian medical papyrus that was written approximately 1550 BC^1^. Until the nineteenth century, it was considered as an incurable disease^2^. By the end of 2021, the American Cancer Society anticipates that around 2 million new cancer cases with 600,000 deaths will occur in the United States^3^.

Phosphatidylinositol-3-kinases (PI3K) are important enzymes that control a variety of physiological processes and have a critical role in apoptosis^4,5^. In tumor tissue and various respiratory inflammations, it is considerably overexpressed^6^. Many malignancies have abnormal PI3K activation, and increased activity also indicates cancer therapy resistance. Unregulated signaling of cancer cells can be caused by a variety of factors, including mutations, tyrosine kinase amplification, or PI3K itself^7,8^. PI3K signaling causes the growth factor to attach to the tyrosine kinase receptor, causing receptor dimerization. As a result, at the internal docking site, the lipid kinase PI3K is activated and initiated. The membrane lipid phosphatidylinositol 4.5-bisphosphate 2 (pip2) is then transformed by PI3K into phosphatidylinositol 4,5-bisphosphate 3 (pip3), an active form of pip2, resulting in the stimulation of the important signaling enzyme protein kinase B (AKT)^9^. AKT promotes cell growth by increasing protein synthesis, which is aided by mTOR signals, and lowers cell death by inhibiting the fork head box o family of proteins (FOXO). They are widely expressed in cells and function to integrate a variety of growth factors, oxidative stress signals, and other stimulatory signals. PI3K indirectly inhibit FOXO which leads to inhibition of apoptosis^8,10^.

Angiogenesis, or the formation of a new vessel from an existing one, is a crucial part of tumor cell proliferation. The angiogenesis process is aided by the presence of VEGF and its receptor (VEGFR). Inhibition of VEGFR is thought to be one of the most promising cancer therapeutic targets^11^. Compared to VEGFR-1 and VEGFR-3, VEGFR-2 has more kinase activity. In VEGF endothelial cells, this receptor is a response controller. These restrictions include permeability, proliferation, invasion, and migration^12^.

VEGFR inhibitors frequently face the challenge of developing of resistance to new therapeutic drugs after a period of treatment, even though antiangiogenetic has proven to be a promising method for cancer therapy. One crucial justification is that numerous additional pathways are turned on when antiangiogenic therapy is administered to reverse the therapeutic effectiveness. In the presence of these antiangiogenic agents, the PI3K signalling pathway, which has been proven to be a bypass or compensation mechanism, might become excessively active^13–15^. Due to tumor hypoxia or oncogene stimulation, PI3Ks increase angiogenic cytokines and modify endothelial cell responses to them. These cytokines promote cell proliferation, migration, differentiation into tubules, and “invasion” of these capillary sprouts into extracellular matrix by signaling through the receptors VEGFR, FGFR, and Tie-2. As a result, stopping the PI3K pathway from becoming activated during antiangiogenesis therapy may reduce tumorigenesis^16–18^.

It is difficult to properly develop dual inhibitors of VEGFRs and PI3Ks since these two kinases belong to different families. A substance that targets both enzymes has not yet been reported. The unavailability of such a substance and the potential for finding dual inhibitors of VEGFR and PI3K with novel scaffolds prompted our investigation.

**Figure 1:**
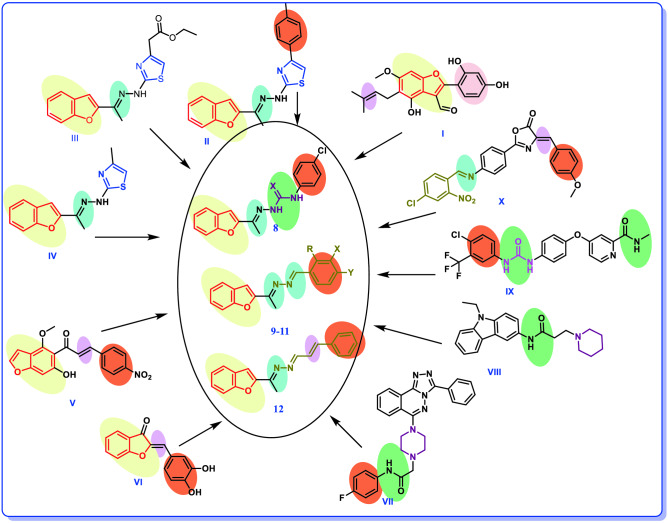
Structures of some reported benzofuran hybrids and PI3K and VEGFR-2 inhibitors, with color map molecular hybridization rational design.

Benzofuran-containing compounds, such as benzofuran III (**I**), are natural substances with substantial anticancer action^19^. In addition, El-Khouly et al. synthesized many new benzofuran derivatives as anticancer agents with significant PI3K inhibition by using molecular hybridization technique. Compounds **II**, **III** and **IV** showed significant anticancer and PI3K inhibitory activity as shown in Fig. 1^20^. Also, the activity of benzofuran derivatives against VEGFR-2 was reported in some studies as compound **V** (IC_50_ = 1.00 × 10^−3^ μM)^21^ and Compound **VI** show high inhibitory activity with IC_50_ = 5.96 μM^22^ (Fig. 1).

Otherwise, some scaffolds have a reported anticancer activity as pieridine, piperazine, benzylidine amide, thiosemicarbazone and semicarbazone.

Compound **VII**, which is a piperazine containing compound, showed a good anticancer activity against Hela, MCF-7 cell lines by IC_50_ values 2.0–3.4 µM respectively^23^ and compound **VIII**, piperidine derivative, that showed a good anticancer activity^24^. In addition, Sorafenib (**IX**) is a urea derivative that approved by the FDA in 2005 for the treatment of advanced renal cancer as VEGFR-2 inhibitor and Compound **X** showed a good activity against different types of this human cancer cell lines (Fig. 1)^25^.

Since molecular hybridization (MH) is a relatively recent concept in the field of drug development^26^ and in continuing of our previous work what mentioned above^20^ it was thought worthwhile to design and synthesis new scaffold of benzofuran hybrids such as benzofuran–pieridine, benzofuran–piperazine, benzofuran–thiosemicarbazone, benzofuran–semicarbazone and benzofuran–benzylidine amide hybrids and assess their anticancer activity using hybridization technique represented in color coded map in Fig. 1. Four cancer cell lines: hepatocellular carcinoma (HePG2), mammary gland breast cancer (MCF-7), epithelioid carcinoma cervix cancer (Hela) and human prostate cancer (PC3) are adopted for the study. Cytotoxicity against human lung fibroblast (WI38) cell line will be assessed to predict their toxicity on normal cell. Further mechanistic studies will be examined on cell cycle distribution and apoptosis in addition to enzymatic inhibitory efficacy on PI3K and VEGFR-2 as a possible target if anticancer mechanism. Moreover, computational studies as molecular docking, surface mapping, contact preference and physicochemical proprieties prediction will be performed to support the biological examination findings.

## Results

### Chemistry

**Figure 2:**
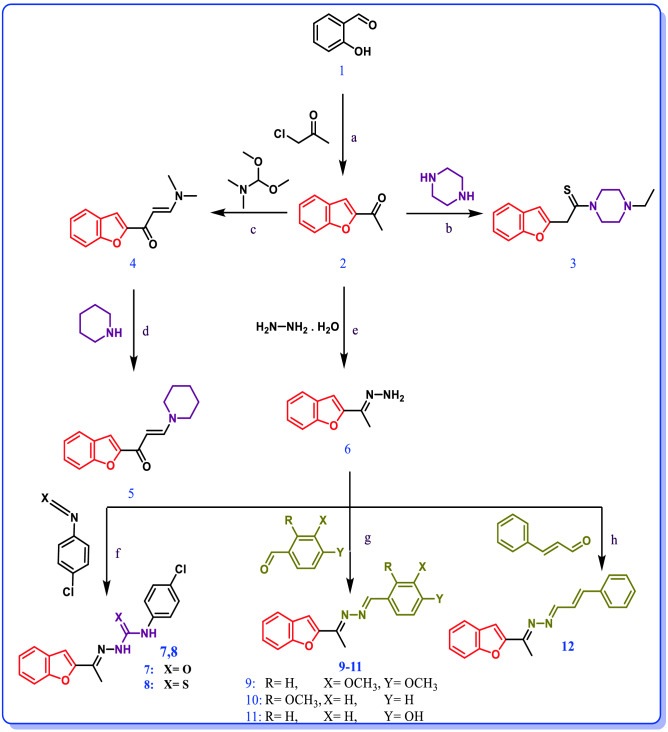
Reaction conditions: (**a**) dry acetone, K_2_CO_3_; (**b**) metallic sulfur, glycerol/K_2_CO_3_ (10:1 (ml/mmol)), 80 °C; (**c**) dry xylene, reflux; (**d**) EtOH, reflux; (**e**) EtOH, reflux; (**f**) THF, rt; (**g**) glacial CH_3_COOH or NH_4_Cl, reflux; and (**h**) glacial CH_3_COOH, reflux.

2-Acetylbenzofuran (**2**) was prepared by condensation of salicylaldehyde and chloroacetone in dry acetone in presence of potassium carbonate (Fig. 2)^27^.

Compound **3** was synthesized by Willgerodt–Kindler’s reaction which is an important reaction in organic synthesis for thioamide formation. This reaction was performed by the reaction of 1-(benzofuran-2-yl)ethan-1-one (**2**), N-ethyl piperazine and metallic sulfur in K_2_CO_3_/glycerol (1:10 (mmol/ml))^28,29^. K_2_CO_3_-glycerol considered as deep eutectic solvent due to its physiochemical properties are significantly different than its primary components in their free states^30^. These properties afford a green environment for running Willgerodt–Kindler’s reaction that it can decrease the temperature needed for the reaction from 130 °C in former works to 80 °C in this work. Also, the glycerol part has a dehumidifying property that can assist the formation of aziridinium intermediate by capturing of released water molecule upon the reaction^28^. Compound **3** was confirmed by ^1^H-NMR with presence of 2 signals at δ: 2.24 and 2.51 ppm from N-ethyl group (Fig. 2).

After that, compound **5** was prepared by addition of piperidine to an ethanolic solution of enaminone **4**^31^ which was produced from the reaction of *N,N*-dimethylformamide dimethyl acetal (DMFDMA) with acetyl benzofuran (**2**)^32^. Compound **5** structure can be confirmed by six proton signals at δ: 1.62 ppm and four proton signal at δ: 3.36 ppm in ^1^H-NMR spectrum (Fig. 2).

Benzofuranyl thiosemicarbazone/semicarbazone hybrids **7**, **8** were prepared by the reaction of appropriate aryl isothiocyanate/isocyanate with hydrazone compound **6** solution in THF at room temperature (Fig. 2)^11^. Compounds **7**, **8** were easily confirmed by the new four proton signals in ^1^H-NMR spectrum at 7.74–7.33 ppm and the two exchangeable protons at 11.04–9.09 ppm.

Benzofuranyl benzylidine amide hybrids **9**–**12** were prepared according to Schiff’s base reaction via condensation reaction of appropriate aryl aldehyde derivative to hydrazone **5** in absolute ethanol to produce desired hybrid (Fig. 2)^33^. The structures of compounds **9**–**12** were confirmed by ^1^H-NMR with singlet signal at δ: 8.91 and 8.94 ppm respectively from methinic proton and new aromatic proton for all compounds. Also, ^13^C-NMR spectral data of compounds displayed carbon signals at δ: 109.68 and 109.71 ppm from 2 (N=C) carbon which confirmed their structure.

### Antitumor screening using MTT assay

**Figure 3:**
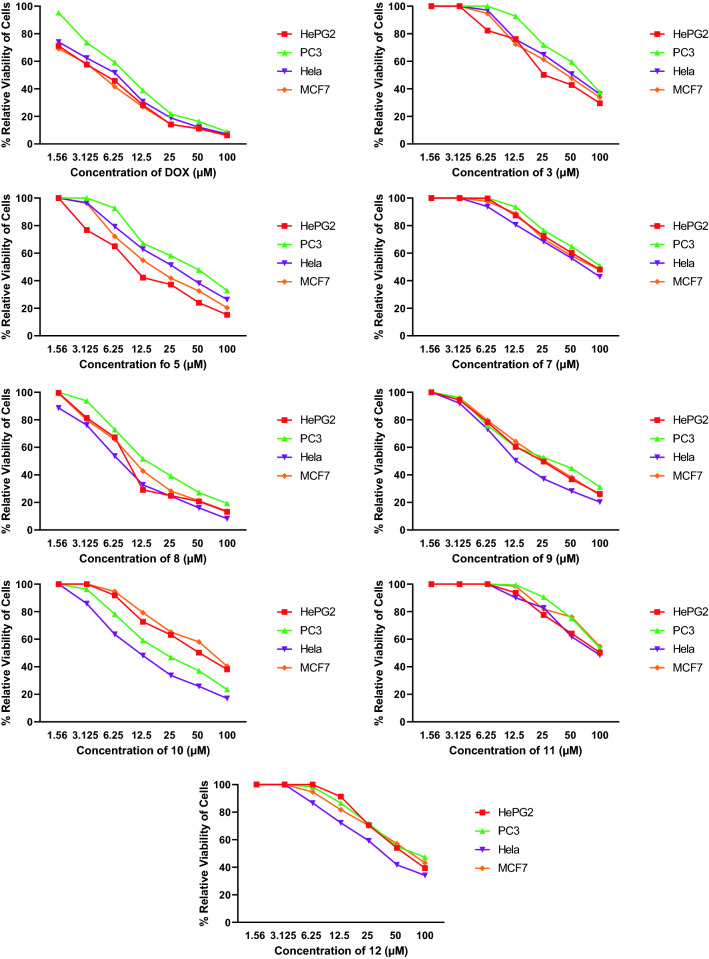
Survival curves of the tested compounds **4**, **5**, **7**–**12**.

Using doxorubicin (DOX) as a reference medication, the newly synthesized compounds were tested against human cancer cell lines. Hepatocellular carcinoma (HePG2), mammary gland breast cancer (MCF-7), epithelioid carcinoma cervix cancer (Hela), and human prostate cancer were included on the panel (PC3). A control cell line, human lung fibroblast (WI38), was utilized to determine the safety margin of the produced chemicals. The cell lines were obtained from the American Type Culture Collection (ATCC) through VACSERA, a Cairo-based holding firm for biological goods and vaccines. The enzyme inhibitory activity of the most active synthesized compound was tested against PI3K and VEGFR-2. After that, the consequence on cell cycle distribution and apoptosis was investigated. Finally, to explain and validate these findings, computational experiments were conducted. The results are summarized in Table 1 and Fig. 3.

**Table 1:** In vitro antitumor activity (IC_50_, µM) of the tested hybrids **3**, **5**, **7**–**12** and DOX.

Comp. no.	In-vitro cytotoxicity IC_50_ (µM)			
	HePG2^a^	MCF-7^b^	Hela^c^	PC3^d^
**3**	34.82 ± 2.5	45.07 ± 2.9	51.37 ± 3.1	65.01 ± 3.2
**5**	12.61 ± 1.0*	19.92 ± 1.5*	28.33 ± 2.2	41.52 ± 2.6
**7**	81.59 ± 3.9	76.91 ± 3.8	66.05 ± 3.2	93.86 ± 4.7
**8**	9.73 ± 0.7 ^ϯ^	11.58 ± 0.9*	7.94 ± 0.5^ϯ^	17.49 ± 1.3*
**9**	26.40 ± 2.1	28.10 ± 2.3	17.02 ± 1.4*	32.10 ± 2.2
**10**	50.97 ± 3.0	62.83 ± 3.3	13.82 ± 1.0*	24.76 ± 1.9*
**11**	84.92 ± 3.2	> 100	89.31 ± 3.2	> 100
**12**	62.04 ± 3.2	68.36 ± 3.6	39.82 ± 2.7	74.38 ± 3.9
**DOX**	4.50 ± 0.2	4.17 ± 0.2	5.57 ± 0.4	8.87 ± 0.6

^a^Hepatocellular carcinoma (HePG2). ^b^Mammary gland breast cancer (MCF-7). ^c^Epithelioid carcinoma cervix cancer (Hela). ^d^Human prostate cancer (PC3).

IC_50_: compound concentration required to inhibit tumor cell proliferation by 50% (mean ± SD, n = 3).

IC_50_ (µM). ^ϯ^1–10 (very strong), *11–25 (strong), 26–50 (moderate), 51–100 (weak), > 100 (non-toxic). *DOX* doxorubicin as a reference drug.

### In vitro cytotoxicity against human normal cell line

The anticancer agent's selectivity for cancer cells is a critical consideration for any researcher, as it can decrease the molecule's adverse effects on normal human cells. As a result, the selectivity to cancer cells is evaluated in this study utilizing the accessible cell line, human lung fibroblast (WI38), as well as doxorubicin (DOX) as a reference drug. All results of in vitro cytotoxicity (IC_50_) of the synthesized compounds against WI38 cell line are summarized in Table 2.

**Table 2:** In vitro cytotoxicity (IC_50,_ µM) of the tested compound and DOX against human normal cell line WI38.

Comp. no.	WI38^a^ IC_50_ (µM)	Comp. no	WI38 IC_50_ (µM)
**3**	78.52 ± 3.8	**9**	48.18 ± 2.8
**5**	63.97 ± 3.1	**10**	67.74 ± 3.4
**7**	> 100	**11**	43.25 ± 2.9
**8**	48.18 ± 2.8	**12**	56.45 ± 3.2
**DOX**	4.50 ± 0.2		

^a^Human lung fibroblast (WI38).

IC_50_: compound concentration required to inhibit normal cell proliferation by 50% (mean ± SD, n = 3).

IC_50_ (µM). ^ϯ^1–10 (very strong), *11–25 (strong), 26–50 (moderate), 51–100 (weak), > 100 (non-toxic). *DOX* doxorubicin as a reference drug.

### Human PI3K enzyme and human VEGFR-2 enzyme inhibitory assay

Compound **8** which is the most active compounds against the four assessed cancer cell lines, their inhibitory effect against human PI3Kα enzyme were tested versus LY294002, potent PI3Kα inhibitory drug^34^, as a reference drug. Compound **8** showed a very strong inhibitory activity against PI3Kα with IC_50_ values 2.21 nM in comparison to 6.18 nM for LY294002 (Table 3).

**Table 3:** IC_50_ values of compound **8** against PI3Kα and VEGFR-2.

Compound	PI3Kα enzymatic IC_50_ (nM)*	VEGFR-2enzymatic IC_50_ (nM)*
**8**	2.21 ± 0.11	86 ± 4
**LY294002**	6.18 ± 0.2	N/A
**Sorafenib**	N/A	34 ± 0.86

*N/A* not applied.

*Mean activity values of triplicate results.

Compound **8** was subjected to assess its inhibitory activity against human VEGFR-2 enzyme relative to the reference compound sorafenib, which a potent VEGFR-2 inhibitory drug^35^. Compound** 8** showed good inhibitory activity against VEGFR-2 with IC_50_ values 86 nM in comparison with sorafenib (IC_50_ = 34 nM) as in Table 3.

### Cell cycle arrest analysis

The integrated results of cytotoxicity and enzyme inhibitory assays put the most active compound into further investigation to make a good understanding for its mechanism of action. So, compound **8** was assessed its activity on cell cycle distribution and induction of apoptosis in different phases of Hela cells.

**Figure 4:**
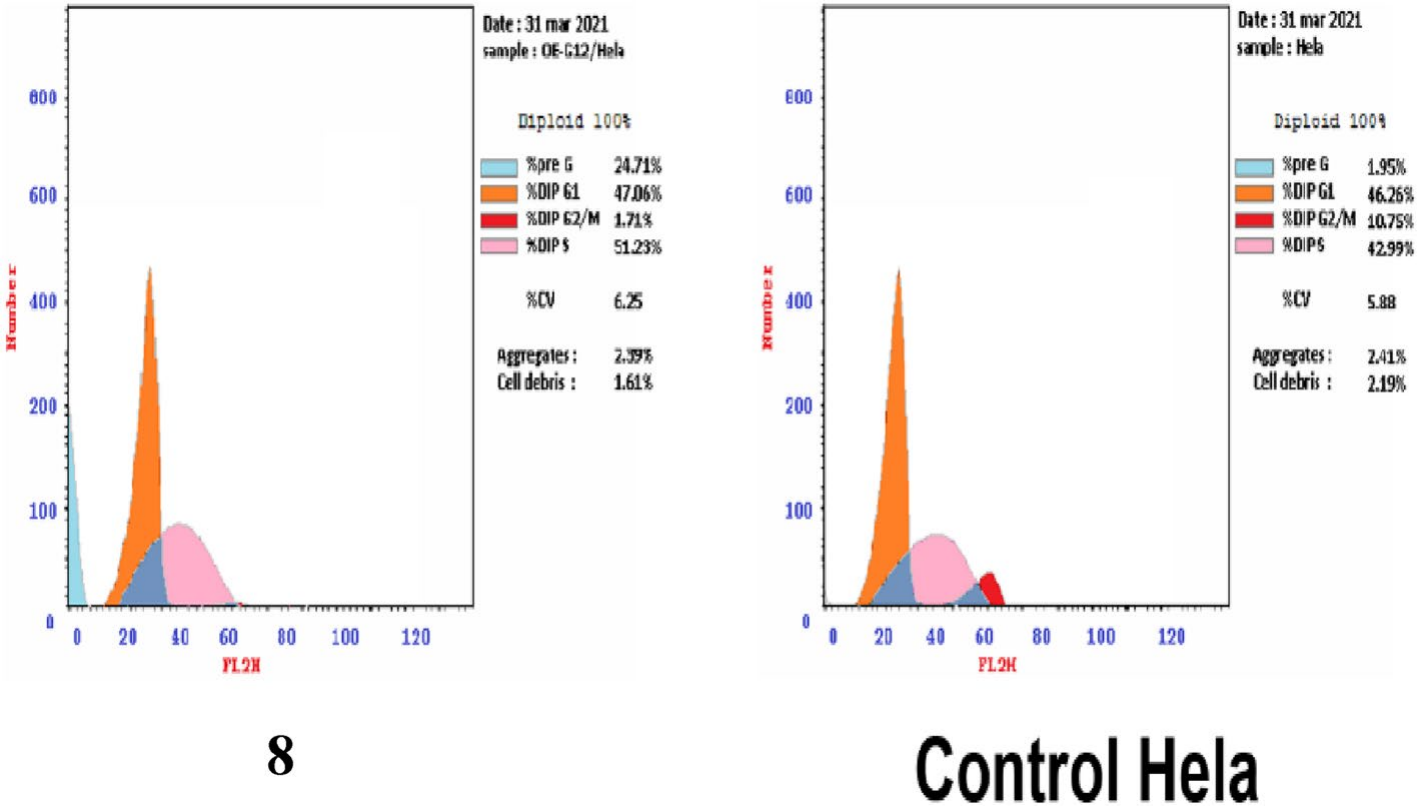
Effect of compound **8** on the cell cycle distribution of Hela cells.

Compound **8** showed that the cell arrested in G1/S phase at 24 h by 51.23% in comparison to control cell (42.99%) (Table 4). The induced apoptosis was determined by assessing the percent of cells blocked in the pre-G1 phase: 24.71% of cells were found in the pre-G1 phase after 48 h exposure of compound** 8** compared to the control cells (1.78% and 1.95%) (Fig. 4).

**Table 4:** Effect of compound **8** on the cell cycle phases in MCF-7 cells.

Compound	Cell cycle distribution (%)				Comment
	%G0–G1	%S	%G2/M	%Pre-G1	
**8/Hela**	47.06	51.23	1.71	24.71	Pre-G1 apoptosis and cell growth arrest at G1/S phase
**Control Hela**	46.26	42.99	10.75	1.95

### Detection of apoptosis

**Figure 5:**
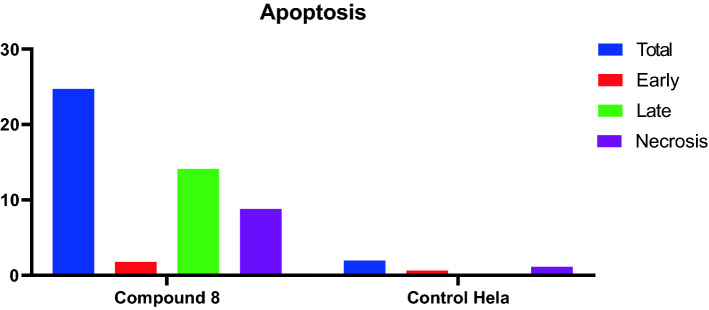
Effect of compound **8** on apoptosis and necrosis in Hela cells.

**Figure 6:**
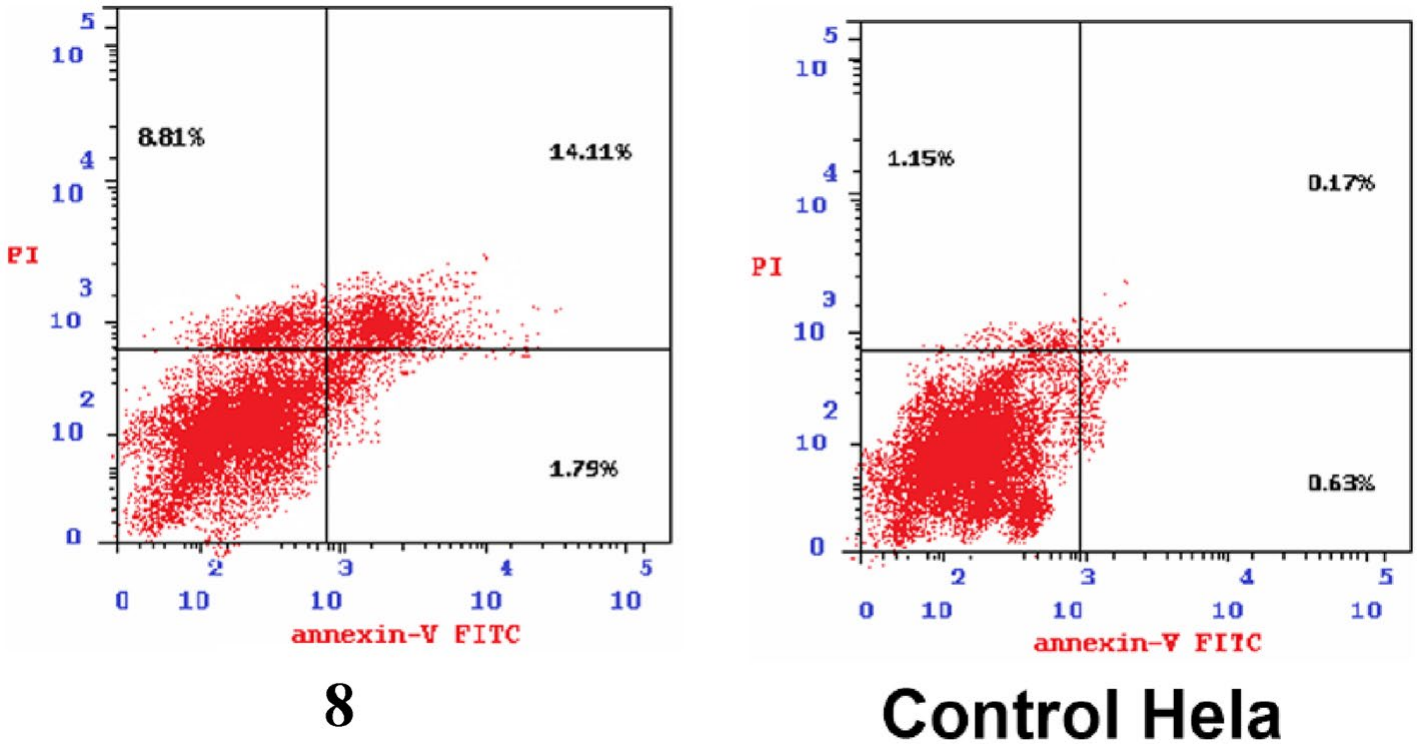
Apoptosis effect on MCF-7 and Hela cell lines induced by compound **8**.

In Hela cells, compound **8** induced an early apoptotic effect with 1.79% at 24 h. Also, the enhanced late apoptotic induction with 14.11% by 83-fold compared to the Hela control cell (0.17%). Moreover, compounds **8** induced apoptosis with 27.7%, the results are shown in Figs. 5 and 6. On other hand, necrotic effect in Hela at 24 h was induced by compounds **8** with 8.81% compared to the control cells (1.15%).

### Computational studies

#### Molecular docking study

The co-crystal structures of the PI3Kα and its binding partner Alpelisib was published in 2013^36^. On the other hand, co-crystal structure of VEGFR-2 and Sorafenib complex was published^37^. Computational docking study was performed to investigate the results of the enzyme inhibition activity of compound **8** against PI3Kα and VEGFR-2. Also, it was aimed to study the interaction between the synthesized compound with PI3Kα and VEGFR-2. These studies were done using Molecular Operating Environment program (MOE).

The first study showed the complex between PI3Kα and Alpelisib was selected as the docking model (PDB code: 4JPS)^36^. Also, LY294002 assessed for its interaction with PI3Kα as shown in Table 5.

**Table 5:** Docking interaction energy of compound **8** against PI3Kα.

Compound	Docking interaction energy (kcal/mol)
**8**	− 7.9
**LY294002**	− 6.5
**Alpelisib**	− 8.1

A comparative computational molecular modeling study between of the highly active compound **8** was performed in comparison to Alpelisib and LY294002. Alpelisib showed a good binding bond through interaction with the hydrogen bond network including three molecules of water and Tyr836, Asp810, Asp933 and Lys802 side chains. Moreover, Lys802 made a hydrogen bond interaction with one fluorine atom of the trifluoromethyl of Alpelisib. Amide group and residue of Gln859 and Val851 performed a pair of donor–acceptor hydrogen bonds.

**Figure 7:**
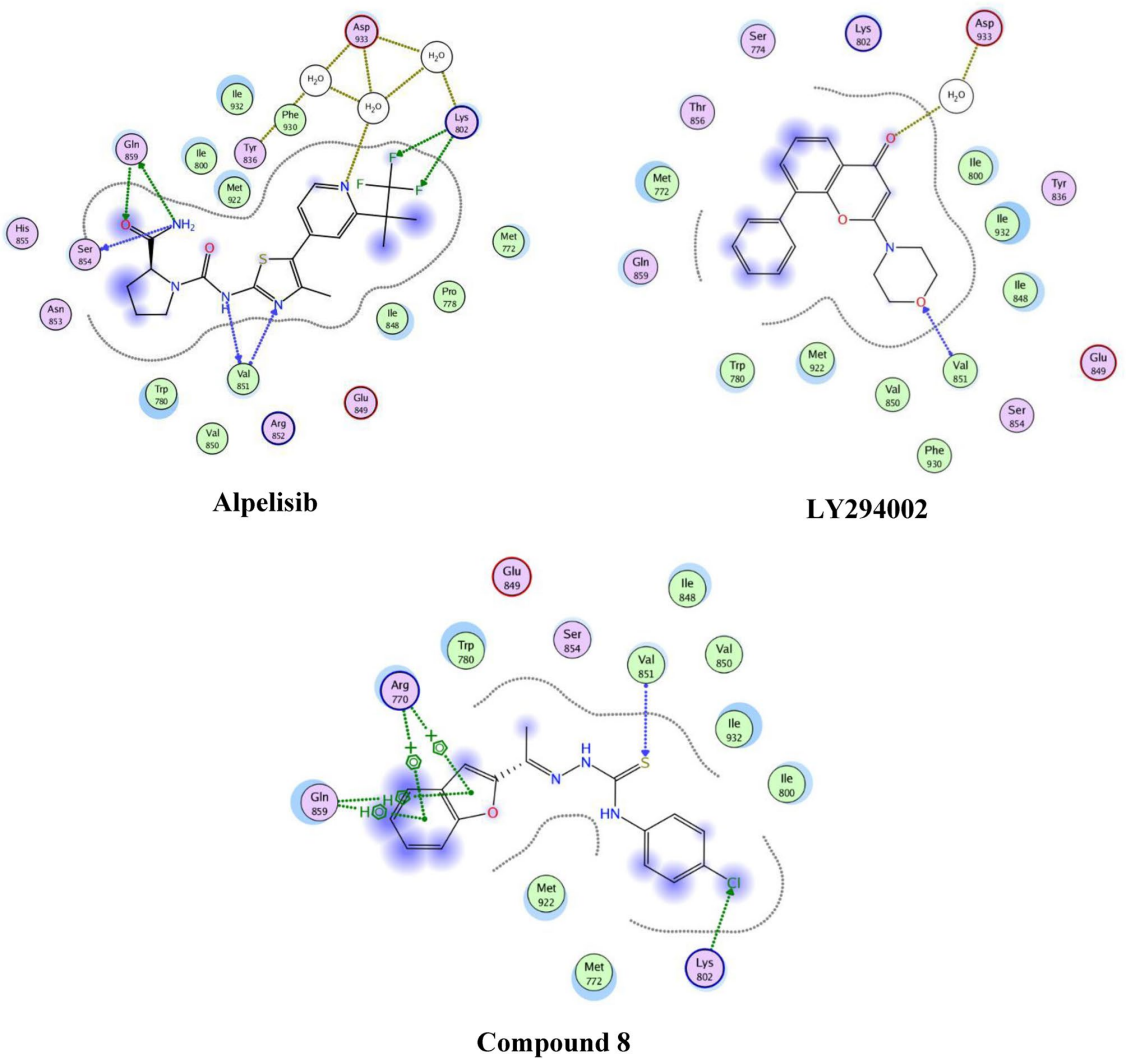
2D binding mode and residues involved in the recognition of compound **8**, alpelisib and LY294002 against PI3Kα.

Compound **8** showed H-bonding with essential Val851 residue and chlorine atom make a H-bond with Lys802. In addition, compound **8** showed interactions with Arg770 and Gln859 with two arene–cation and two arene–H interactions respectively (Fig. 7).

The second study showed the complex between VEGFR-2 and sorafenib was selected as the docking model (PDB code: 3WZE)^37^ as shown in Table 6.

**Table 6:** Docking interaction energy of compound **8** against VEGFR-2.

Compound	Docking interaction energy (kcal/mol)
**8**	− 7.0
**Sorafenib**	− 8.1

**Figure 8:**
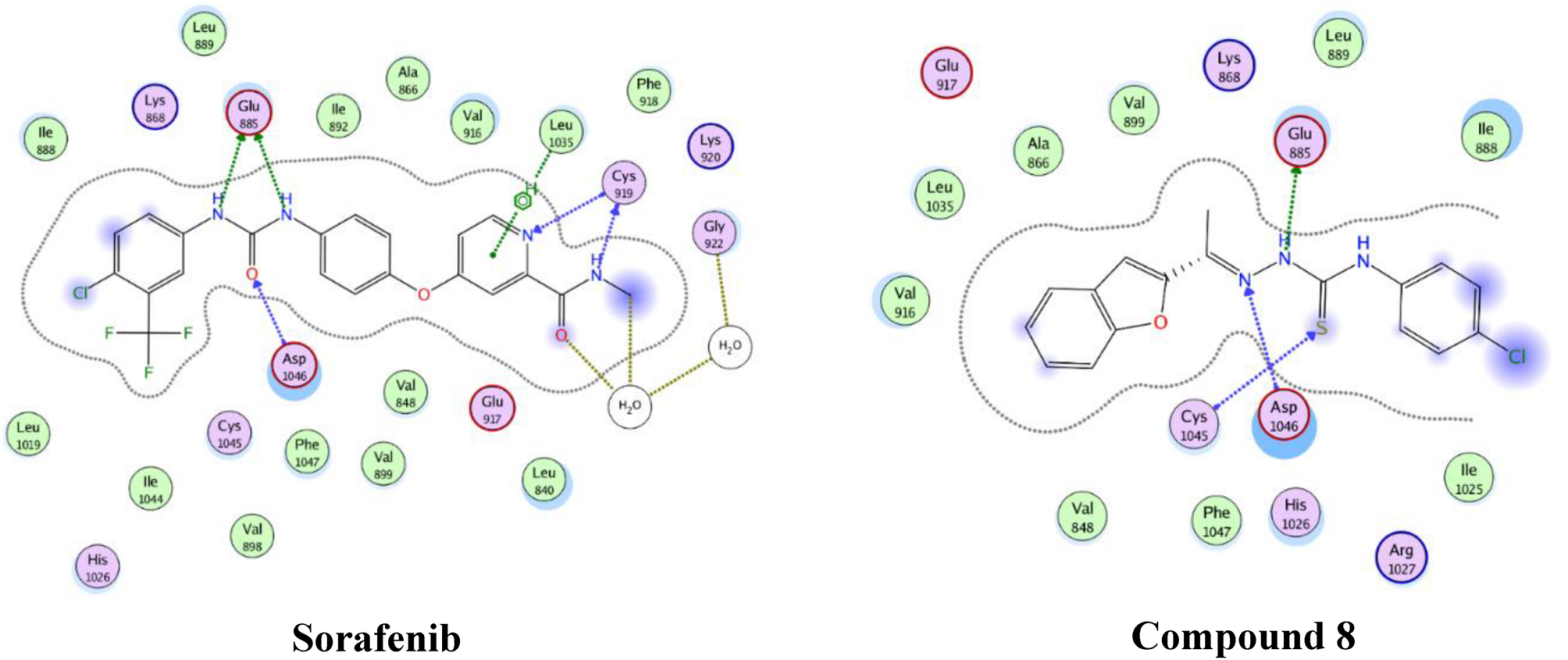
2D binding mode and residues involved in the recognition of compound **8** and sorafenib against VEGFR-2.

Compound **8** showed H-bonding Glu885, Cys1045 and Asp1046. These interactions were subjected to explain them activity and the 2D interactions were illustrated in Fig. 8.

#### Surface mapping

**Figure 9:**
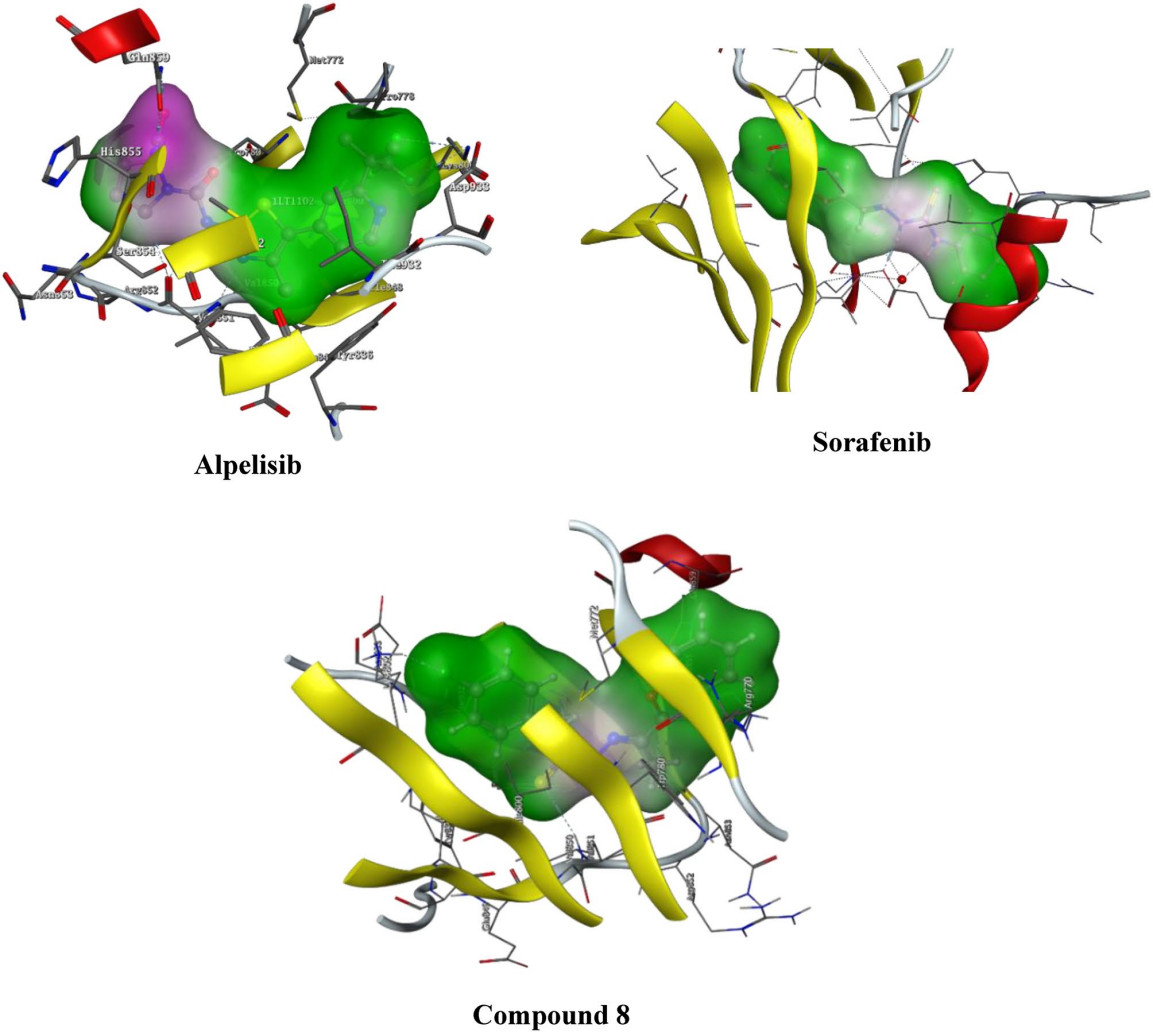
3D surface map for alpelisib, sorafenib and compound **8**. Pink: hydrophilic, white: neutral, green hydrophobic.

**Figure 10:**
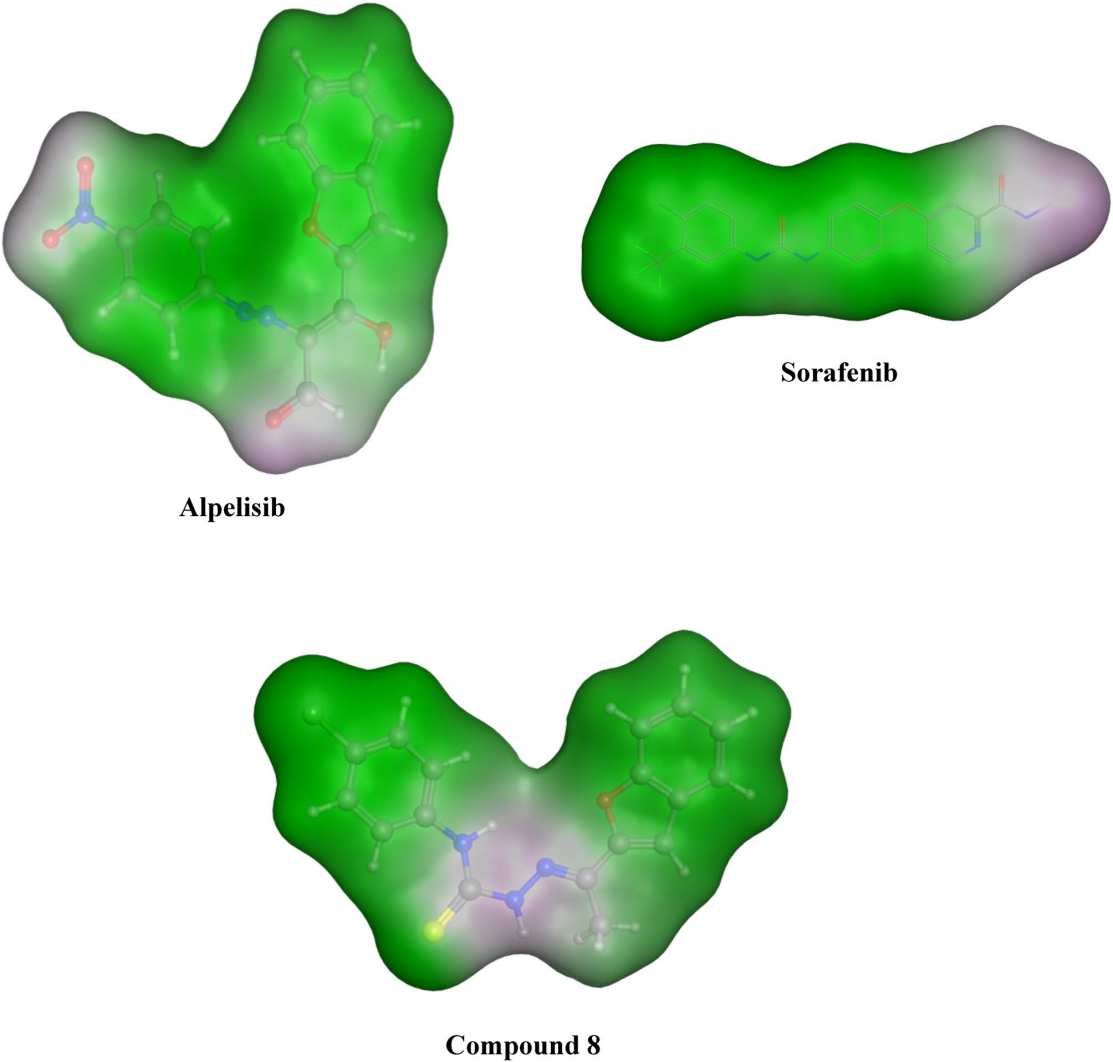
Surface map for alpelisib, sorafenib and compound **8**. Pink: hydrophilic, white: neutral, green hydrophobic.

Surface mapping is a second evaluation phase that confirms the binders of the most active chemicals are comparable to those of the PI3K and VEGFR-2 enzymes. Compound **8** and alpelisib then compound **8** and sorafenib, when combined with a certain enzyme, provide almost normal surface mapping contours. Furthermore, this might provide a reasonable explanation for their excellent binding to a specific enzyme and provide additional evidence that their activity is influenced by PI3K and VEGFR-2 inhibition, respectively (Figs. 9 and 10).

#### Contact preference

**Figure 11:**
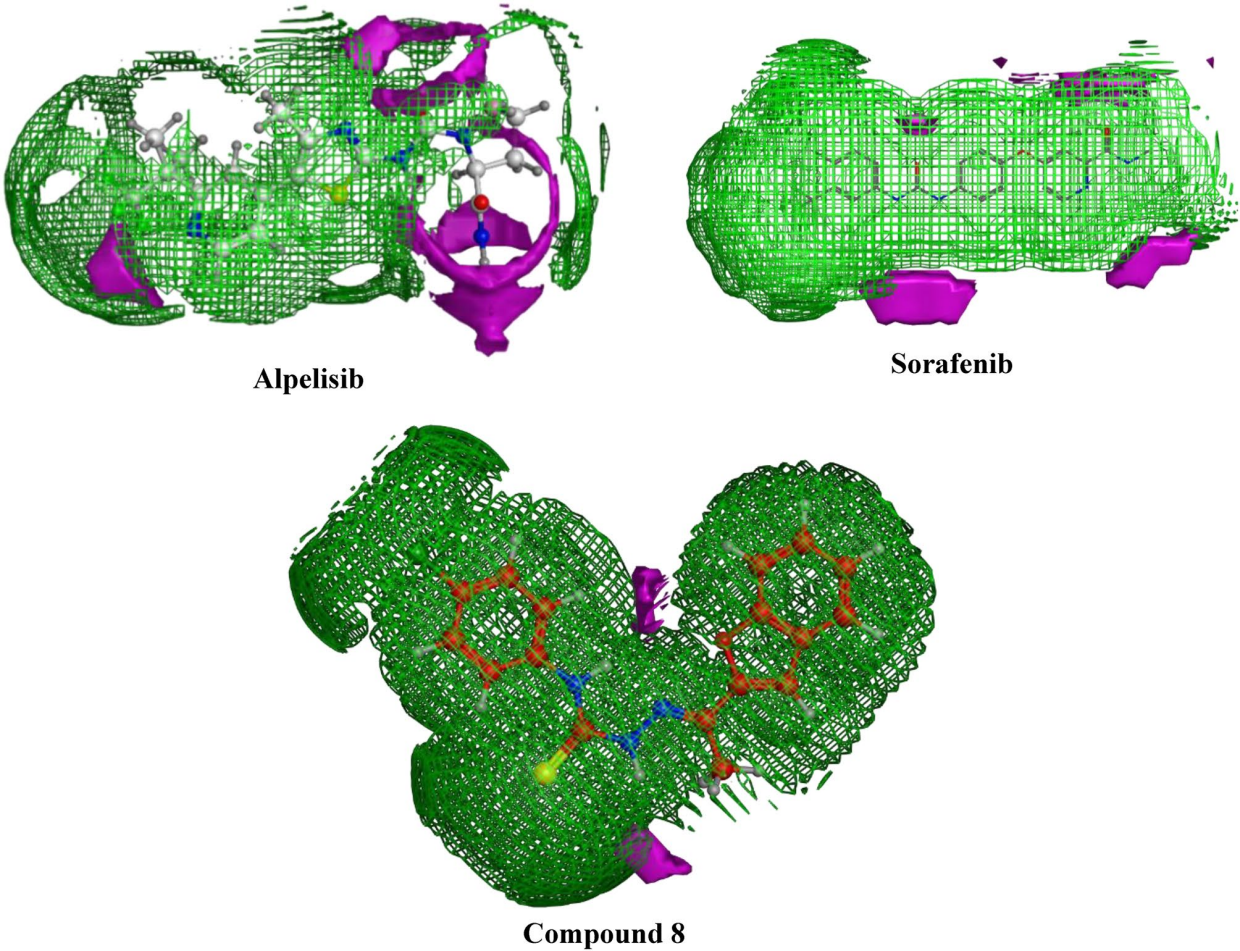
Contact preference for compound **8** and alpelisib. Hydrophobic: green, hydrophilic: red.

Contact statistics application aim is to calculate, from the 3D atomic coordinates of ligand, the best positions for hydrophobic and hydrophilic ligand atoms. Moreover it includes the interactions between the chemical components of the ligands and the protein microenvironment surrounding them. The results obtained illustrates the fragment contacts is valuable since it clearly shows the similarity pattern of distribution of the hydrophobic and hydrophilic sites between our ligands, **8** against the two reference candidates as shown in (Fig. 11). This will lead to A better understanding of its high activity patterns against the two selected enzymes PI3K and VEGFR-2.

#### Physicochemical properties and Lipinski's rule of five

Oral bioavailability is an important criterion for development of active molecules as therapeutic candidates. Molecular characters such as membrane permeability and bioavailability is always associated with some basic molecular descriptors such as hydrogen bond acceptors and donors logP (partition coefficient), molecular weight. Additional criteria such as topological polar surface area (TPSA) and number of rotatable bonds (nRB) were added later. The obtained results revealed that most active synthesized compound **8**, with relation to Alpelisib, ensures a good compliance with the Lipinski rule of five. The SwissADME web service^38^ was used to make the calculations. Table 7 shows the ADME characteristics predicted for the produced substances. From these data, it could be suggested that compound **8** had good drug-likeness with acceptable physicochemical properties.

**Table 7:** Solubility, topological surface area and calculated Lipinski's rule of five for compound **8** and Alpelisib.

Comp	Log *S*^a^	TPSA^b^	MW^c^	M log *P*^d^	nRB^e^	nHBA^f^	nHBD^g^	nVio^h^
**8**	− 5.12	81.65	343.83	2.99	5	2	2	0
**Alpelisib**	− 4.42	129	441.5	2.95	4	8	2	0

^a^Solubility parameter.

^b^Topological polar surface area (Å^2^).

^c^Molecular weight (g/mol).

^d^Lipophilicity parameter.

^e^Number of rotatable bonds.

^f^Number of hydrogen bond acceptors.

^g^Number of hydrogen bond donor.

^h^Number of violations to Lipinski's rule of five.

## Discussion

In this study, new benzofuran derivatives has been synthesized. ^1^H-NMR, ^13^C-NMR, IR, and elemental analyses were used to confirm the structures of the produced compounds. The anticancer activity of the newly synthesized compounds was tested in vitro against HePG, MCF-7, Hela, and PC3 cell lines. Benzofuranyl piperazine hybrid **3** showed weak anticancer activity towards the four cells but the hybridization between pieridine and benzofuran can increase the activity as compound **5** showed strong activity towards HePG2 and MCF-7 cell lines with IC_50_ value 12.61 and 19.92 µM respectively. In addition, benzofuranyl thiosemicarbazone **8** showed very strong inhibition activity against HePG2, and Hela cell lines with IC_50_ values 9.73 and 7.94 respectively but benzofuranyl semicarbazone **7** showed weak activity toward all assessed cell lines. Otherwise, benzofuranyl benzylidine amide hybrids **9**–**12** showed no anticancer activity towards the most of tested cell lines except **9** and **10** showed strong to moderate activity against Hela and PC3 cell line with IC_50_ values range of 13.82–32.10 µM and it indicates that methoxy substitution can increase the anticancer activity.

However, the WI38 cell line was used to test the safety index in vitro and the most active compound **8** (IC_50_ = 48.18 µM) is significantly less toxic than DOX towards WI38 in comparison to DOX (IC_50_ = 6.72 µM). Compound **7** is the least toxic anticancer compound versus all assessed compounds.

**Figure 12:**
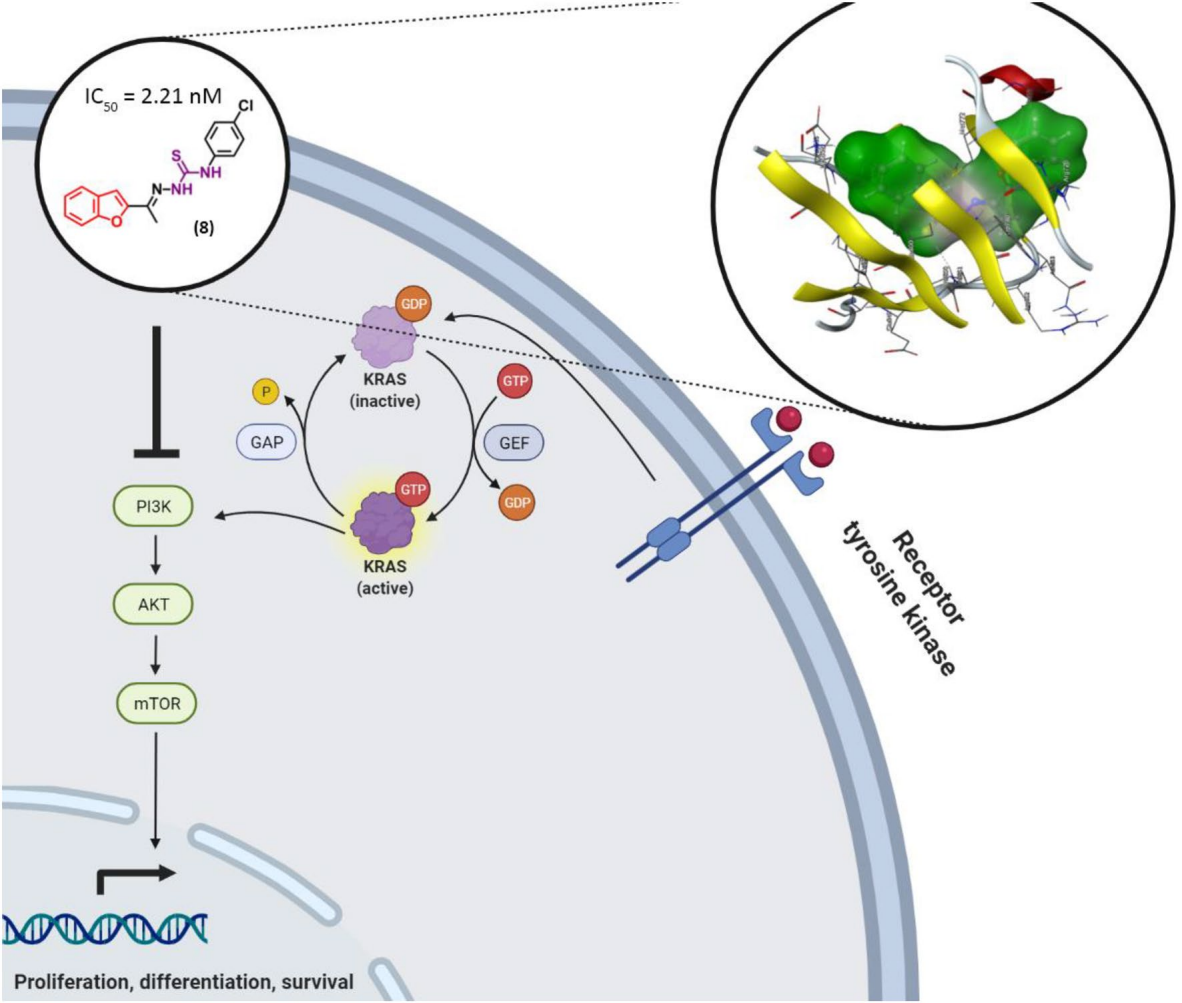
Illustration of inhibitory mechanism of compound **8** on PI3K enzyme.

The activity of compound **8** against the four-tested cancer cells showed an excellent matching with the enzymatic inhibition of PI3Kα which is parallel with the calculated docking interaction energy. These results strongly suggested that the PI3Kα inhibitory mechanism might be one of the main mechanisms of action of the anticancer activity of compound **8** with very strong activity against PI3Kα. The proposed mechanism of action of compound **8** is illustrated in Fig. 12 (created with BioRender.com).

**Figure 13:**
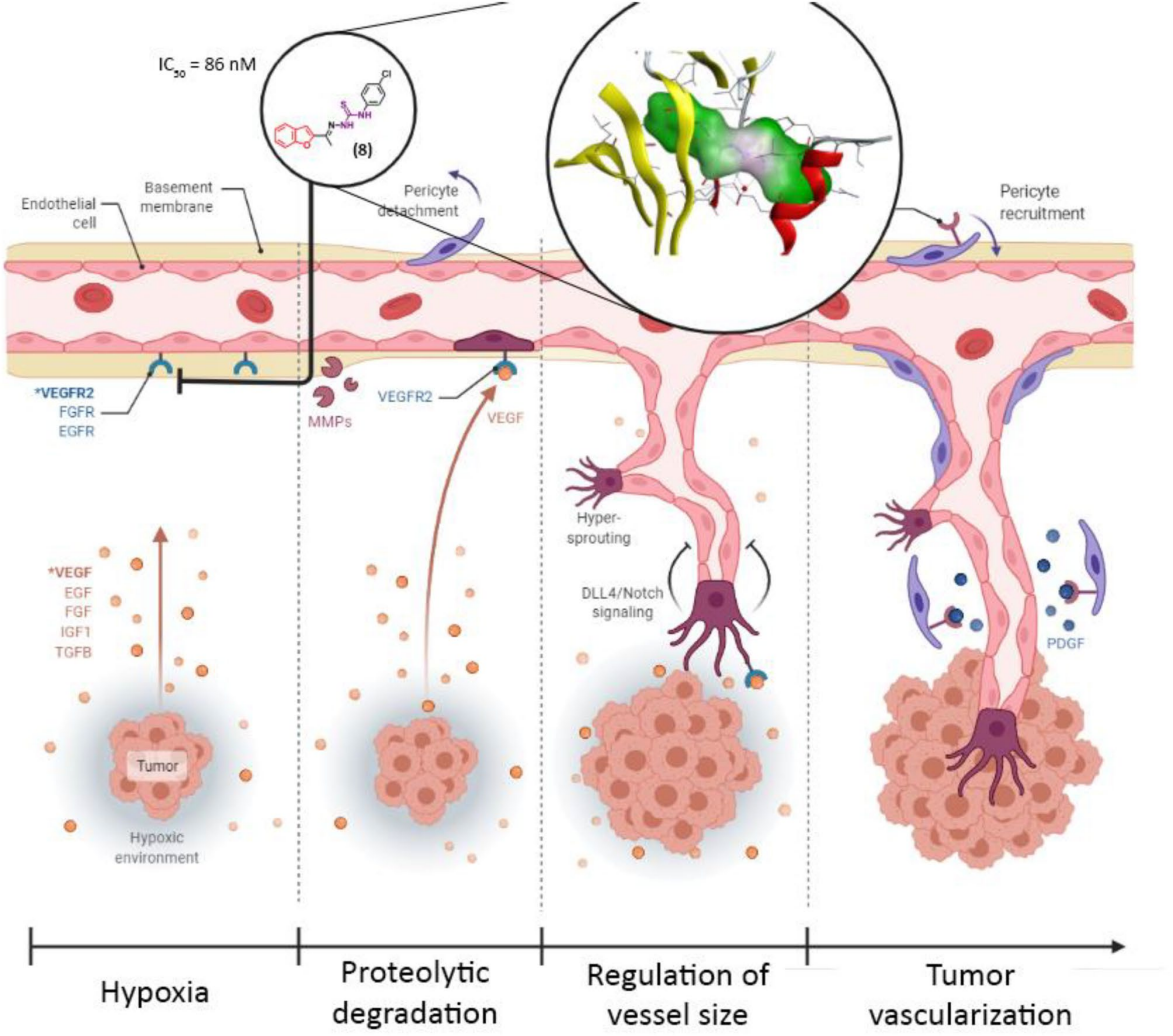
Illustration of inhibitory mechanism of compound **8** on VEGFR-2 enzyme.

Also, VEGFR-2 inhibitory activity results strongly suggested that the VEGFR-2 inhibitory mechanism might be one of modes of action of the anticancer activity of compound **8**. The proposed mechanism of action of compound **8** is illustrated in Fig. 13 (created with BioRender.com).

In comparison, compound **8** inhibited PI3K and VEGFR-2 with IC_50_ values of 2.21 and 68 nM, respectively. In compared to 6.18 nM for compound LY294002, a PI3K potent medication, and 31.2 nM for compound sorafenib, a VEGFR-2 potent drug, compound **8** displayed PI3K and VEGFR-2 inhibition with IC50 values of 2.21 and 68 nM, respectively. As a PI3K and VEGFR-2 inhibitor, the molecular docking and binding affinity of the produced compounds were approximated and investigated computationally using molecular operating environment software (MOE).

The findings of Physicochemical properties and Lipinski's rule of five study revealed that compound **8** follows Lipinski's rule in the same way that Alpelisib does, implying that compound 8 has drug-like characteristics. Predictions of water solubility of compound **8** and Alpelisib revealed that compound 8 has a mostly comparable solubility to Alpelisib; however, compound 8 has a topological polar surface area (TPSA) of 81.65, compared to Alpelisib (TPSA = 129).

In conclusion, the design, synthesis and biological evaluation of new benzofuran hybrids targeting Cancer were performed. The structure of the prepared compounds was confirmed by IR, ^1^H NMR, ^13^C NMR. Anti-proliferative activity of newly synthesized compounds was evaluated against panel of 4 cell lines. The results demonstrated that benzofuranyl thiosemicarbazone 8 showed very strong inhibition activity against HePG2, and Hela cell lines with IC_50_ values 9.73 and 7.94 respectively. Compound 8 showed higher activity/selectively against hepatocellular and cervical carcinoma cell lines. Compound 8 also inhibited PI3K and VEGFR-2 with IC_50_ values of 2.21 and 68 nM, respectively, compared to 6.18 nM for compound LY294002 and 31.2 nM for compound sorafenib as PI3K and VEGFR-2 reference inhibitors, selectively. Different molecular modeling techniques supports the results found. Physicochemical properties assures that compound 8 could be used as a lead to future optimization. Further studies targeting hepatocellular carcinoma and cervical cancer.

## Methods

### Chemistry

The melting points (in °C) were measured with a Stuart melting point apparatus and are uncorrected. Thermo Fisher SCIENTIFIC Nicolet IS10 Spectrometer (v in cm^−1^) was used to record IR spectra (KBr) at Mansoura University's Faculty of Pharmacy. At the NMR facility, Faculty of Pharmacy, Mansoura University, Egypt, ^1^H-NMR and ^13^C-NMR spectra were acquired in a BRUKER 400 MHz using TMS as an internal standard (chemical shifts in ppm, units). Thin layer chromatography (TLC) plates with Silica gel 60 F254 precoated (E. Merck) were used to monitor reaction completion, and the spots were seen using UV light (366 nm). Sigma-Aldrich provided all of the compounds, which were utilized without additional purification. Compounds **2**^27^, **4**^32^ and **6**^39^ were synthesized according to the previously reported procedures.

#### Synthesis of 2-(benzofuran-2-yl)-1-(4-methylpiperazin-1-yl)ethanethione (3)

At 80 °C, a mixture of 1-(benzofuran-2-yl)ethan-1-one (**2**) (0.16 g, 1 mmol), 1-ethylpiperazine (0.11 g, 1 mmol) and metallic sulfur (0.32 g, 10 mmol) was stirred for 2 h in glycerol/K_2_CO_3_ (10:1 (ml:mmol), 25 ml). Following completion of the reaction, the reaction mixture was poured into ice/water, and the product was extracted using ethyl acetate and cleaned using column chromatography (ethyl acetate:petroleum ether, 1:1) to yield the desired compound. Brown solid, m.p. = 125–127 °C, Yield = 70%. ^1^H NMR (400 MHz, CDCl_3_): δ = 7.92 (s, 1H), 7.89 (d, *J* = 8 Hz, 1H), 7.78 (d, *J* = 8 Hz, 1H), 7.61 (t, *J* = 7.5 Hz, 1H), 7.42 (t, *J* = 7.5 Hz, 1H), 4.27–4.20 (m, 2H), 3.67 (t, *J* = 4.5 Hz, 2H), 3.38 (s, 2H), 2.57 (t, *J* = 4.5 Hz, 2H), 2.51 (t, *J* = 4.5 Hz, 2H), 2.38 (t, *J* = 4.5 Hz, 2H), 2.28–2.23 (m, 3H); ^13^C-NMR (100 MHz, CDCl_3_): δ = 191.83, 177.36, 156.16, 149.74, 129.90, 127.11, 124.96, 124.61, 118.42, 112.88, 54.75, 53.91, 51.87, 47.26, 45.49; Elemental analysis for C_15_H_18_N_2_OS, calculated: C, 65.66; H, 6.61; N, 10.21. Found: C, 65.86; H, 6.41; N, 10.11.

#### Preparation of 1-(benzofuran-2-yl)-3-(dimethylamino)prop-2-en-1-one (4)^20^

Golden yellow solid, m.p. = 129–131 °C, Yield = 90%. ^1^H-NMR (400 MHz, DMSO-d_6_): δ = 7.81 (d, *J* = 12.4 Hz, 1H), 7.74 (d, *J* = 7.7 Hz, 1H), 7.66 (d, *J* = 8.3 Hz, 1H), 7.56 (s, 1H), 7.43 (t, *J* = 7.5 Hz, 1H), 7.30 (t, *J* = 7.5 Hz, 1H), 5.85 (d, *J* = 12.4 Hz, 1H), 3.17 (s, 3H), 2.94 (s, 3H) ^13^C-NMR (100 MHz, DMSO-d_6_): δ = 176.56, 155.90, 154.84, 154.491, 128.04, 127.03, 123.91, 123.07, 112.29, 109.17, 91.47, 45.12, 37.70.

#### Synthesis of (E)-1-(benzofuran-2-yl)-3-(piperidin-1-yl)prop-2-en-1-one (5)

A mixture of 1-(benzofuran-2-yl)-3-(dimethylamino) prop-2-en-1-one (**4**) (0.215 g, 1 mmol) and piperidine (0.085 g, 1 mmol) was refluxed in ethanol overnight. The reaction mixture poured in ice/water and the solid formed is filtered then recrystallized from aqueous ethanol. Bale brown solid, m.p. = 136–138 °C, yield = 70%. ^1^H-NMR (400 MHz, CDCl_3_): δ = 77.81 (d, *J* = 12.4 Hz, 1H), 7.58 (d, *J* = 7.6 Hz, 1H), 7.47 (d, *J* = 8.3 Hz, 1H), 7.33 (s, 1H), 7.30 (d, *J* = 7.2 Hz, 1H), 7.23–7.13 (m, 1H), 5.91 (d, *J* = 12.6 Hz, 1H), 3.36 (s, 4H), 1.62 (s, 6H). ^13^C-NMR (100 MHz, CDCl_3_): δ = 178.69, 155.74, 155.15, 152.95, 128.08, 126.44, 123.28, 122.54, 112.03, 108.93, 90.97, 55.33, 46.63, 26.53, 25.05, 24.04. Elemental analysis for C_16_H_17_NO_2_, calculated: C, 75.27; H, 6.71; N, 5.49. Found: C, 77.75; H, 5.62; N, 4.98.

#### Preparation of (E)-[1-(benzofuran-2-yl)ethylidene]hydrazine (6)^39^

In ethanol, 1-(Benzofuran-2-yl)ethan-1-one (**2**) (1.6 g, 10 mmol) was refluxed for 2 h with hydrazine hydrate 99% (0.5 g, 0.5 ml, 10 mmol) The white precipitate was filtered before being crystallized from ethanol. White solid m.p. = 80 °C, yield = 90%.

#### General procedure of synthesis of (E)-2-[1-(benzofuran-2-yl)ethylidene]-*N*-(4-chlorophenyl)hydrazine-1-carbamide (7, 8)

A solution of hydrazone **7** (0.17 g, 1 mmol) in THF (10 ml) was reacted with appropriate isocyanate/isothiocyanate (1.2 mmol) and stirred at room temperature. Upon completion of the reaction as judged by TLC, the reaction mixture was poured on crushed ice. The precipitated solid was filtered, washed with water, and dried. The product was crystallized from ethanol to urea/thiourea derivatives.

#### (E)-2-[1-(benzofuran-2-yl)ethylidene]-*N*-(4-chlorophenyl)hydrazine-1-carboxamide (7)

Using 4-chlorophenyl isocyanate (0.18 g, 1.2 mmol). White solid, m.p. = 235–237 °C, Yield = 90%. ^1^H-NMR (400 MHz, DMSO-d_6_): δ = 10.18 (s, exchangeable H, NH), 9.09 (s, exchangeable H, NH), 7.75 (d, *J* = 8.7 Hz, 2H), 7.73 (d, *J* = 6.0 Hz, 1H), 7.68 (d, *J* = 8.2 Hz, 1H), 7.58 (s, 1H), 7.42 (d, *J* = 8.7 Hz, 2H), 7.39 (t, *J* = 7.4 Hz, 1H), 7.33 (t, *J* = 7.4 Hz, 1H), 2.35 (s, 3H). ^13^C-NMR (100 MHz, DMSO-d_6_): δ = 155.69, 154.86, 145.46, 138.70, 132.62, 129.18, 129.18, 128.54, 128.18, 125.38, 123.50, 123.25, 120.93, 120.93, 113.21, 102.93, 13.64; Elemental analysis for C17H14ClN3O2, calculated: C, 62.30; H, 4.31; N, 12.82. Found: C, 64.10; H, 3.94; N, 11.56.

#### (E)-2-(1-(benzofuran-2-yl)ethylidene)-*N*-(4-chlorophenyl)hydrazine-1-carbothioamide (8)

Using 4-chlorophenyl isothiocyanate (0.20 g, 1.2 mmol). Yellow solid, m.p. = 207–209 °C, Yield = 70%. ^1^H-NMR (400 MHz, DMSO-d_6_): δ = 11.04 (s, exchangeable H, NH), 10.17 (s, exchangeable H, NH), 7.74 (d, *J* = 8.4 Hz, 2H), 7.68 (t, *J* = 7.6 Hz, 3H), 7.49 (d, *J* = 8.6 Hz, 2H), 7.42 (t, *J* = 7.5 Hz, 1H), 7.33 (t, *J* = 7.4 Hz, 1H), 2.47 (s, 3H); ^13^C-NMR (100 MHz, DMSO-d_6_): δ = 177.40, 153.96, 141.28, 138.47, 128.53, 127.78, 126.21, 123.93, 122.16, 111.93, 107.71, 14.30; Elemental analysis for C_17_H_14_ClN_3_OS, calculated: C, 59.39; H, 4.10; N, 12.22. Found: C, 58.76; H, 5.25; N, 13.12.

#### General procedure for the preparation of compounds (9–12)

With a catalytic quantity of glacial acetic acid or ammonium chloride, a combination of hydrazone **7** (0.17 g, 1 mmol) and suitable benzaldehyde derivative (1 mmol) was refluxed in absolute ethanol (25 ml) for 3–24 h. The solvent was concentrated and filtered before being recrystallized from ethanol to obtain the desired compound.

#### (E)-1-[1-(benzofuran-2-yl)ethylidene]-2-((E)-3,4-dimethoxybenzylidene)hydrazine (9)

Using 3,4-dimethoxybenzaldehyde (0.17 g, 1 mmol) with catalytic amount of ammonium chloride (0.05 g). Pale yellow solid, m.p. = 130–132 °C, yield = 80%. ^1^H-NMR (400 MHz, DMSO-d_6_): δ = 8.55 (s, 1H), 7.76 (d, *J* = 7.7 Hz, 1H), 7.70 (t, *J* = 7.3 Hz, 1H), 7.59 (d, *J* = 4.4 Hz, 1H), 7.55 (s, 1H), 7.44 (t, *J* = 7.4 Hz, 1H), 7.35 (s, 1H), 7.33 (d, *J* = 7.5 Hz, 1H), 7.10 (d, *J* = 7.5 Hz, 1H), 3.85 (s, 6H), 2.38 (s, 3H); ^13^C-NMR (100 MHz, DMSO-d_6_): δ = 159.83, 156.19, 155.28, 154.02, 153.85, 152.20, 151.60, 149.47, 128.25, 127.42, 126.86, 124.01, 122.56, 112.00, 111.95, 109.96, 56.12, 55.93, 14.99; Elemental analysis for C_19_H_18_N_2_O_3_, calculated: C, 70.79; H, 5.63; N, 8.69 Found: C, 70.56; H, 5.78; N, 8.46.

#### (E)-1-[1-(benzofuran-2-yl)ethylidene]-2-((E)-2-methoxybenzylidene)hydrazine (10)

Using 2-methoxybenzaldehyde (0.14 g, 1 mmol) with catalytic amount of ammonium chloride (0.05 g). Yellow powder, m.p. = 154–156 °C, yield = 85%. ^1^H-NMR (400 MHz, DMSO-d_6_): δ = 8.82 (s, 1H), 8.04 (d, *J* = 6.7 Hz, 1H), 7.76 (d, *J* = 7.7 Hz, 1H), 7.71 (d, *J* = 8.2 Hz, 1H), 7.61 (s, 1H), 7.45 (t, *J* = 7.7 Hz, 1H), 7.34 (t, *J* = 7.5 Hz, 1H), 7.18 (d, *J* = 7.3 Hz, 1H), 7.08 (t, *J* = 7.1 Hz, 2H), 3.91 (s, 3H), 2.52 (s, 3H); ^13^C-NMR (100 MHz, DMSO-d_6_): δ = 155.28, 153.86, 151.59, 128.25, 126.86, 123.99, 122.57, 112.04, 109.71, 14.99; Elemental analysis for C_18_H_16_N_2_O_2_, calculated: C, 73.95; H, 5.52; N, 9.58, Found: C, 72.95; H, 6.52; N, 8.58.

#### (E)-1-[1-(benzofuran-2-yl)ethylidene]-2-((E)-4-hydroxybenzylidene)hydrazine (11)

Using 4-hydroxybenzaldehyde (0.12 g, 1 mmol) with catalytic amount of glacial acetic acid. Yellow powder, m.p. = 154–156 °C, yield = 85%. ^1^H-NMR (400 MHz, DMSO-d_6_): δ = 9.68 (s, 1H), 8.94 (s, 1H), 8.73 (s, 1H), 8.38 (d, *J* = 8.1 Hz, 1H), 8.33 (d, *J* = 7.7 Hz, 1H), 7.83 (t, *J* = 7.9 Hz, 1H), 7.75 (d, *J* = 7.7 Hz, 1H), 7.69 (d, *J* = 8.2 Hz, 1H), 7.59 (s, 1H), 7.43 (t, *J* = 7.6 Hz, 1H), 7.31 (d, *J* = 7.4 Hz, 1H), 2.37 (s, 3H); ^13^C-NMR (100 MHz, DMSO-d_6_): δ = 155.20, 153.66, 151.42, 130.97, 128.17, 126.96, 124.04, 122.631, 116.30, 111.95, 109.80, 14.93; Elemental analysis for C_17_H_14_N_2_O_2_, calculated: C, 73.37; H, 5.07; N, 10.07, Found: C, 71.54; H, 7.22; N, 11.72.

#### (1E,2E)-1-[1-benzofuran-2-yl) ethylidene]-2-((E)-3-phenylallylidene)hydrazine (12)

Using 3-phenylprop-2-enal (0.13 g, 1 mmol) with catalytic amount of glacial acetic (0.05 ml) acid. Golden solid, m.p. = 141–143 °C, yield = 85%. ^1^H-NMR (400 MHz, DMSO-d_6_): δ = 8.41 (d, *J* = 9.5 Hz, 1H), 7.72–7.65 (m, 3H), 7.48–7.36 (m, 6H), 7.31 (s, 1H), 7.20 (d, *J* = 9.5 Hz, 1H), 7.16 (d, *J* = 9.6 Hz, 1H), 2.52 (s, 3H); ^13^C-NMR (100 MHz, DMSO-d_6_): δ = 195, 163.70, 153.75, 143.86, 136.08, 131.73, 130.01, 129.57, 129.43, 129.24, 129.00, 128.02, 125.80; Elemental analysis for C_19_H_16_N_2_O, calculated: C, 79.14; H, 5.59; N, 9.72. Found: C, 78.75; H, 6.21; N, 8.92.

### Antitumor screening and using MTT assay in vitro cytotoxicity against human normal cells

All the newly synthesized compounds were evaluated against human cancer cell lines using doxorubicin (DOX) as a reference drug. The panel consisted of hepatocellular carcinoma (HePG2), mammary gland breast cancer (MCF-7), epithelioid carcinoma cervix cancer (Hela) and human prostate cancer (PC3). Human lung fibroblast (WI38) was used to assay the cytotoxicity effect on normal cell. The cell lines were obtained from ATCC via Holding company for biological products and vaccines (VACSERA), Cairo, Egypt. The cytotoxic activity was defined as the concentration of the compound that causes 50% growth inhibition compared with the growth of untreated cells.

### Human PI3Kα and VEGFR-2 enzyme inhibition assay

PI3Kα and VEGFR-2 activity were verified by enzyme-linked immunosorbent assay (ELISA) assay technique. The assay exploited a specific antibody for human PI3Kα and VEGFR-2 firmly coated on a 96-well plate, in which 100 ml of the standard solution or the tested compounds were added to every well at room temperature. Then, the mean absorbance of each group of standard and tested compounds was determined. The standard curve was drawn on log–log paper with the absorbance on the Y-axis and the standard concentration on the X-axis. Percent Inhibition was assessed through comparing both test compounds and control results, whereas IC_50_ was assessed from concentration/inhibition curve using LY294002 and sorafenib as standards.

### Cell cycle arrest analysis and apoptosis detection

Annexin V-FITC and PI apoptosis kit were used to detect apoptosis (eBioscience™, San Diego, CA, USA). On a six well plate, Mcf cells were plated at a density of 600,000 cells/ml. The cells were treated with compound 8 at 10 mg/ml after 24 h of incubation. The solvent control consisted of cells developed in medium containing an equivalent volume of DMSO. In accordance with the instructions supplied by the Annexin V-FITC and PI apoptosis kit, the cells were stained with an Annexin V-FITC conjugate and propidium iodide (PI) after 24 h. The percentage of apoptotic, necrotic, and alive cells was then calculated. By using a flow cytometer (NovoCyte, ACEA Biosciences Inc, San Diego, CA, US) and the NovoExpress 1.3.0 software (ACEA Biosciences Inc, San Diego, CA, US), the cells' released fluorescence was examined. Each point on the population plot ("dot plot") corresponds to a single event with a particular fluorescence signal in relation to the axes: Annexin V-FITC green fluorescence.

### Computational studies

The three-dimensional structures of selected substituted benzofuran which represent the best anticancer activity, in their neutral forms, were built by using the MOE of Chemical Computing Group Inc. software 2014. The Lowest energy conformers of new analogues ‘global-minima’ was docked into the binding pocket of the pdb models 4JPS^36^ and 3WZE^37^. It was obtained from the Protein Data Bank of Brookhaven National Laboratory. The hydrogens were added, then enzyme structure was subjected a refinement protocol where the constraints on the enzyme were gradually removed and minimized until the RMSD gradient was 0.01 kcal/mol Å. Energy minimization was performed using the molecular mechanics force field ‘AMBER.’ For each benzofuran derivative, energy minimizations (EM) were performed using 1000 steps of steepest descent, followed by conjugate gradient minimization to a RMSD energy gradient of 0.01 kcal/mol Å. The active site of the enzyme was detected in reference compound pdb file. The compounds under study underwent flexible alignment experiment using ‘Molecular Operating Environment’ software (MOE of Chemical Computing Group Inc., on a Core i5 2.40 GHz workstation)^40^. The molecules were constructed using the Builder module of MOE. Their geometry was optimized by using the MMFF94 force field followed by a flexible alignment using systematic conformational search. The Lowest energy aligned conformers were identified. For each analogue, the partial atomic charges were assigned using the semi-empirical mechanical calculation “AM1” method implemented in the program. Conformational search was done. All the conformers were minimized until the RMSD deviation was 0.01 kcal/mol, then subjected to surface mapping, color coded: pink, hydrogen bond; white: neutral; green hydrophobic.

Lipophilicity “log P” and the polar surface area (Å^2^) “TPSA” for the tested analogues were estimated using SWISS ADME web service (http://www.swissadme.ch).

## Supplementary Information

Supplementary Information.

## Data Availability

All data generated or analyzed during this study are included in this published article [and its supplementary information files].
