# Supplementary material for: Design, synthesis and computational study of new benzofuran hybrids as dual PI3K/VEGFR2 inhibitors targeting cancer
Source: Sci Rep. 2022 Oct 12;12:17104. doi: 10.1038/s41598-022-21277-2 (PMC9556824; doi:10.1038/s41598-022-21277-2)
Supplement: Supplementary file 1 — Supplementary Information. [file 41598_2022_21277_MOESM1_ESM.pdf]

2-(benzofuran-2-yl)-1-(4-methylpiperazin-1-yl)ethanethione ***(3)***.

^{1}HNMR

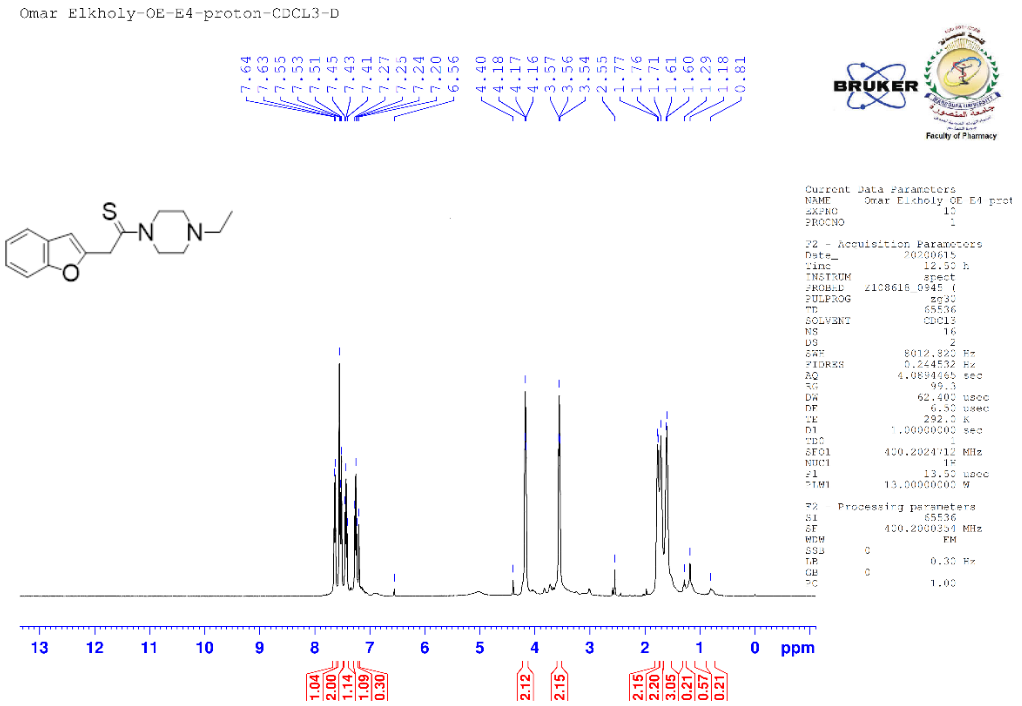

^{13}CNMR

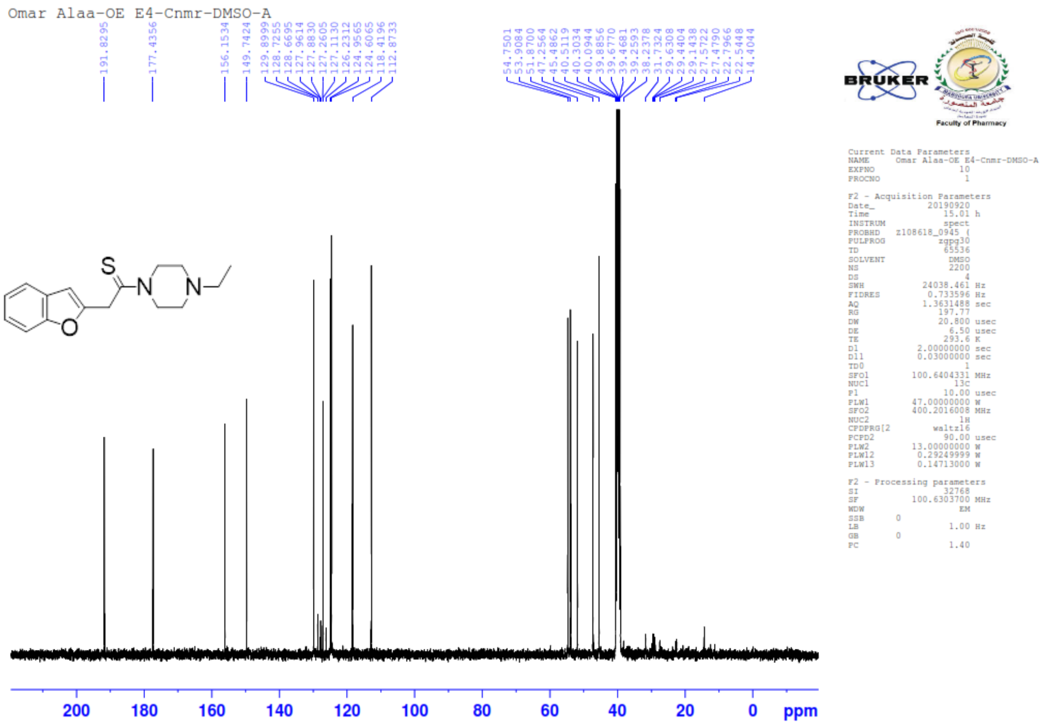

(E)-1-(benzofuran-2-yl)-3-(piperidin-1-yl)prop-2-en-1-one **(5)**

^{1}HNMR

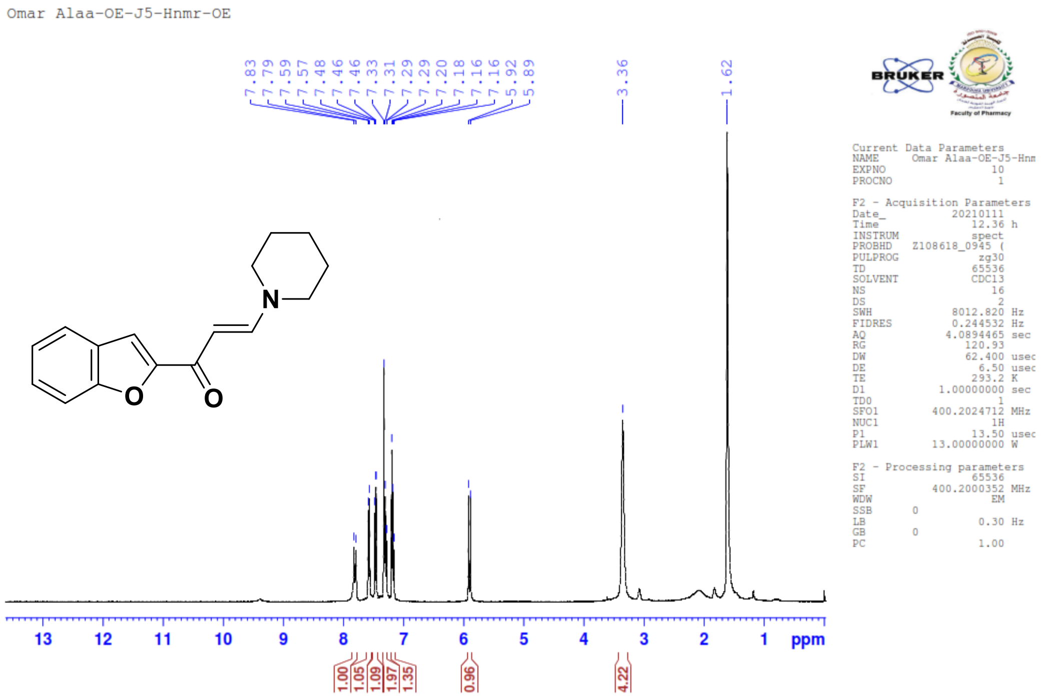

^{13}CNMR

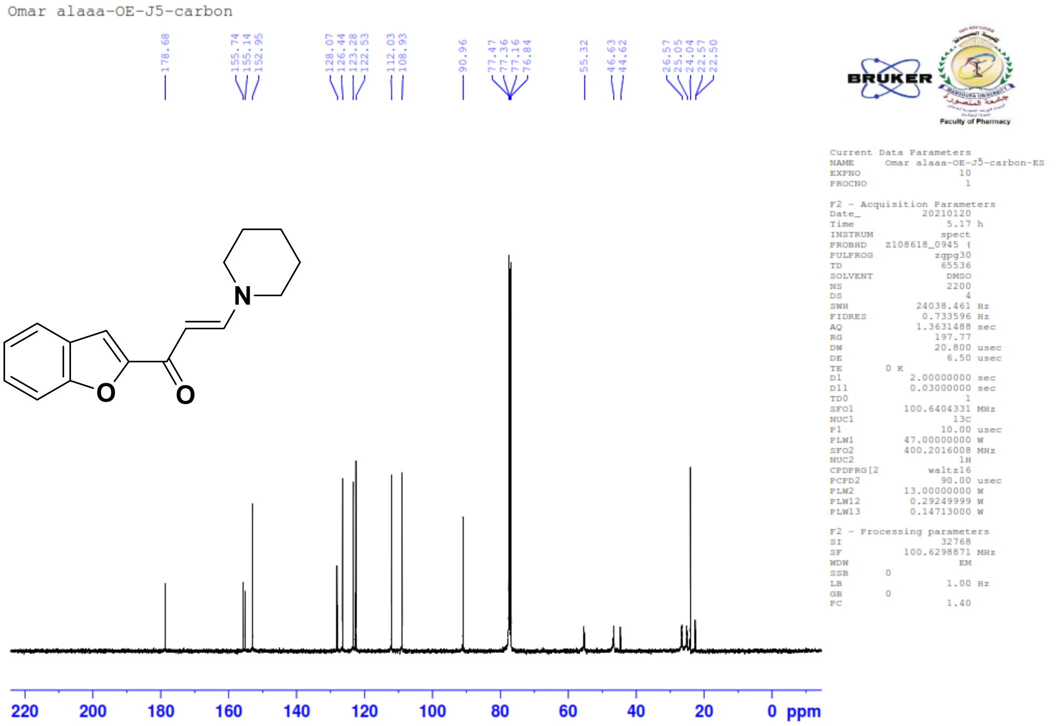

(E)-2-[1-(benzofuran-2-yl)ethylidene]-N-(4-chlorophenyl)hydrazine-1-carboxamide ***(7)***

^{1}HNMR

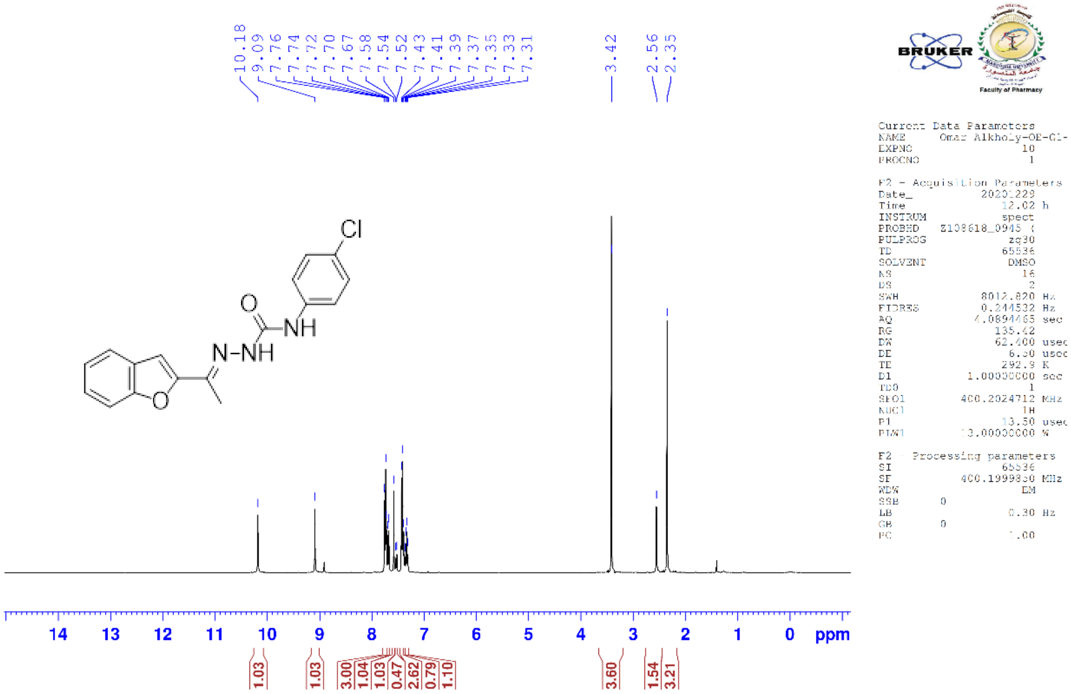

^{13}CNMR

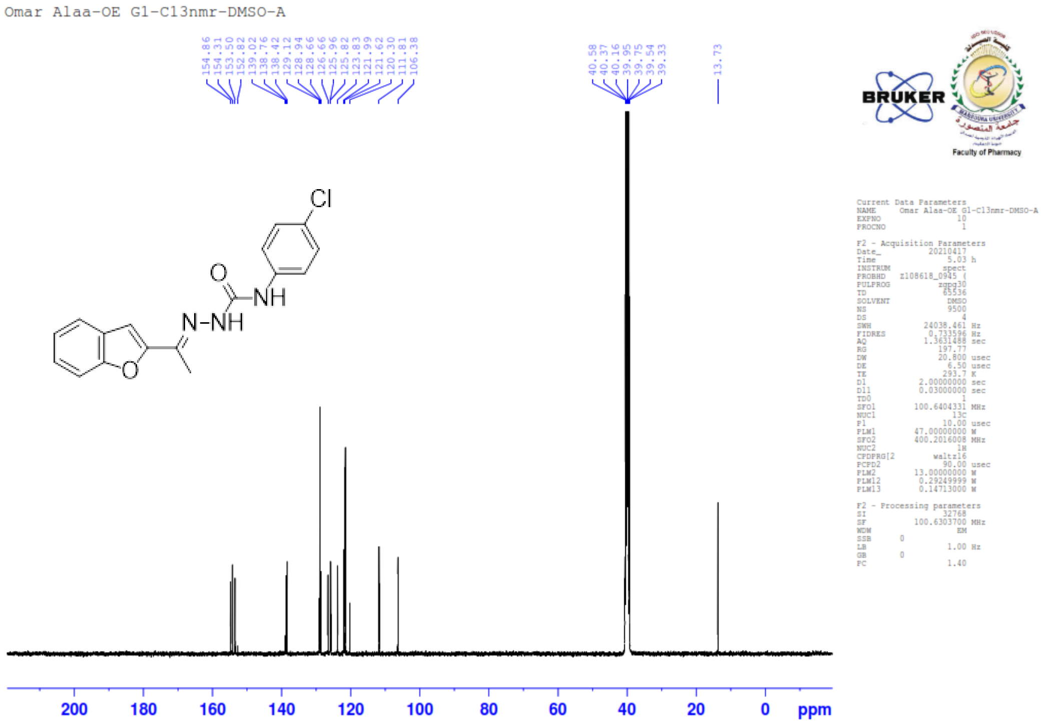

(E)-2-(1-(benzofuran-2-yl)ethylidene)-N-(4-chlorophenyl)hydrazine-1-carbothioamide ***(8)***

^{1}HNMR

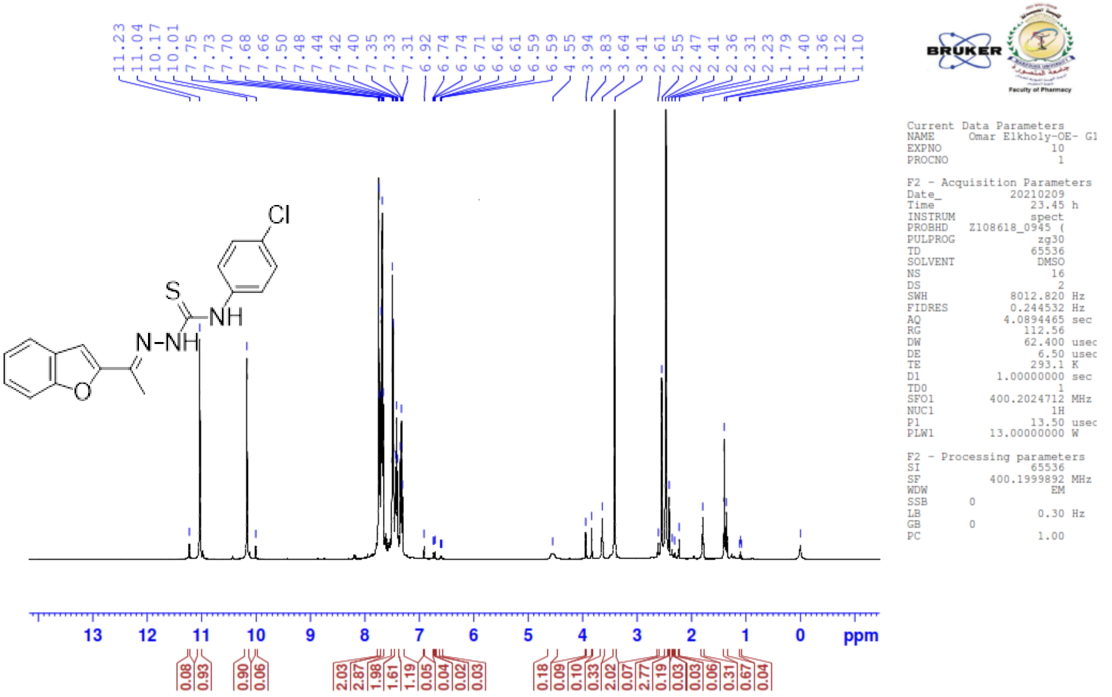

^{13}CNMR

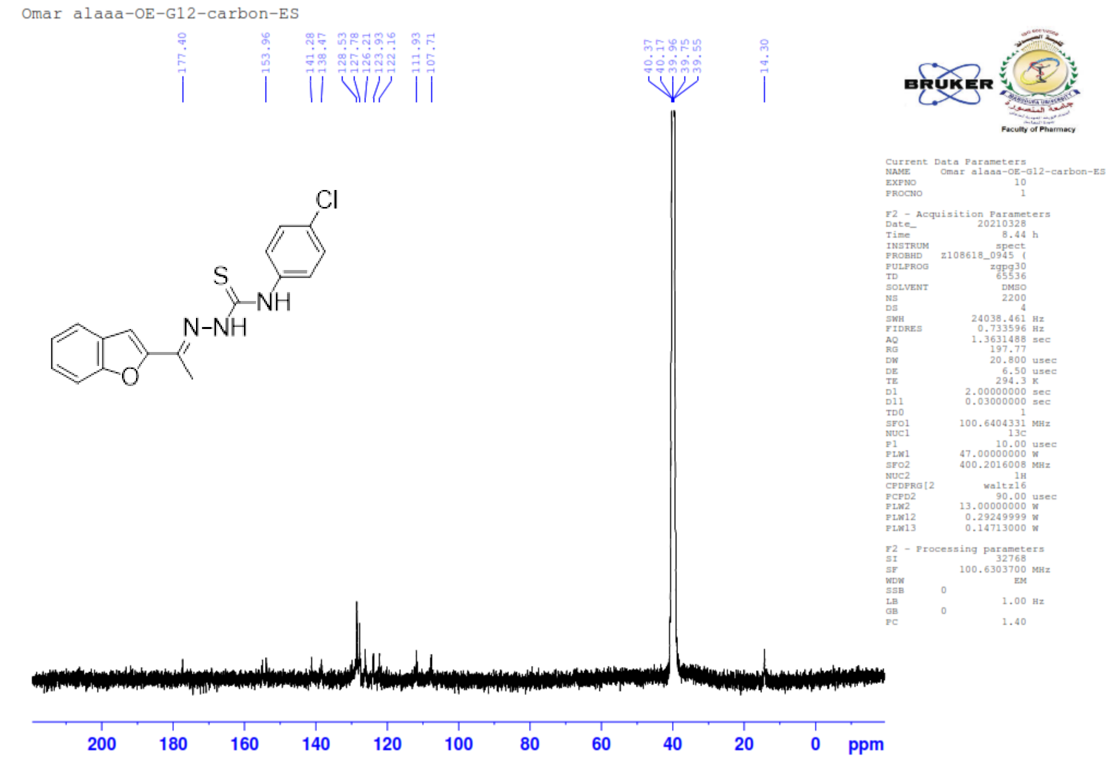

(E)-1-[1-(benzofuran-2-yl)ethylidene]-2-((E)-3,4-dimethoxybenzylidene)hydrazine **(9)**

^{1}HNMR

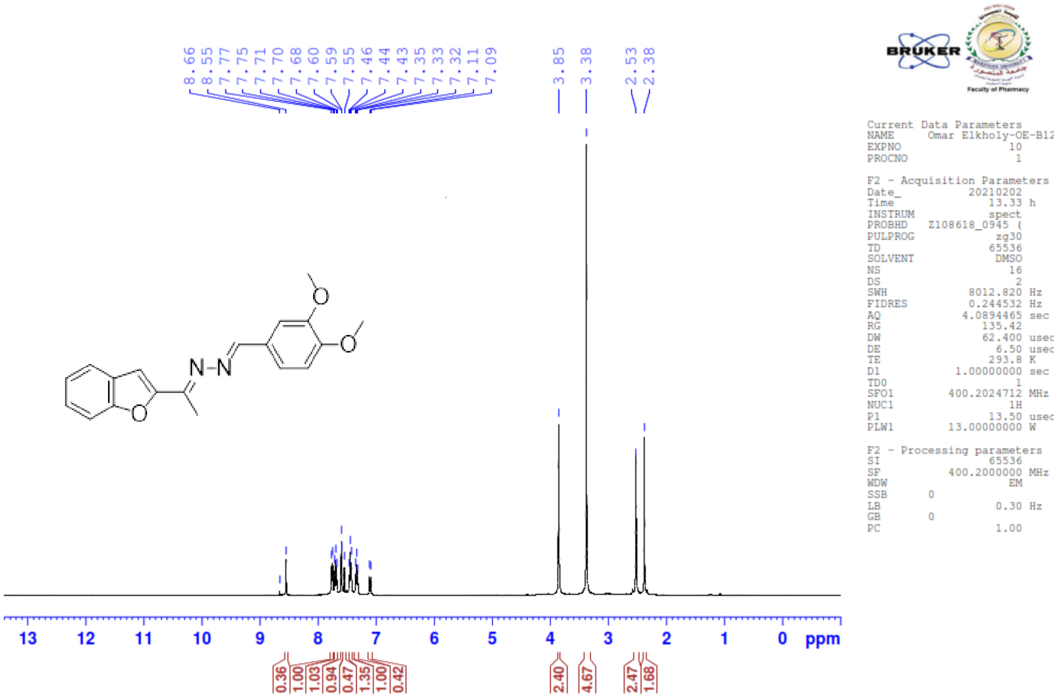

^{13}CNMR

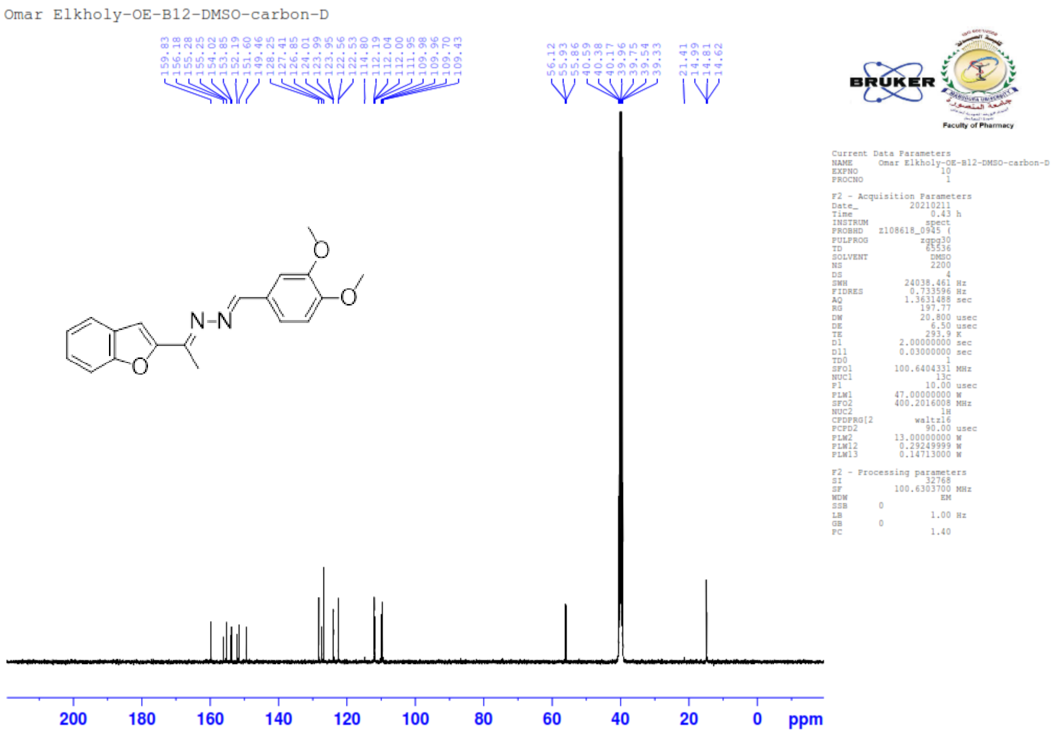

(E)-1-[1-(benzofuran-2-yl)ethylidene]-2-((E)-2-methoxybenzylidene)hydrazine ***(10)***

^{1}HNMR

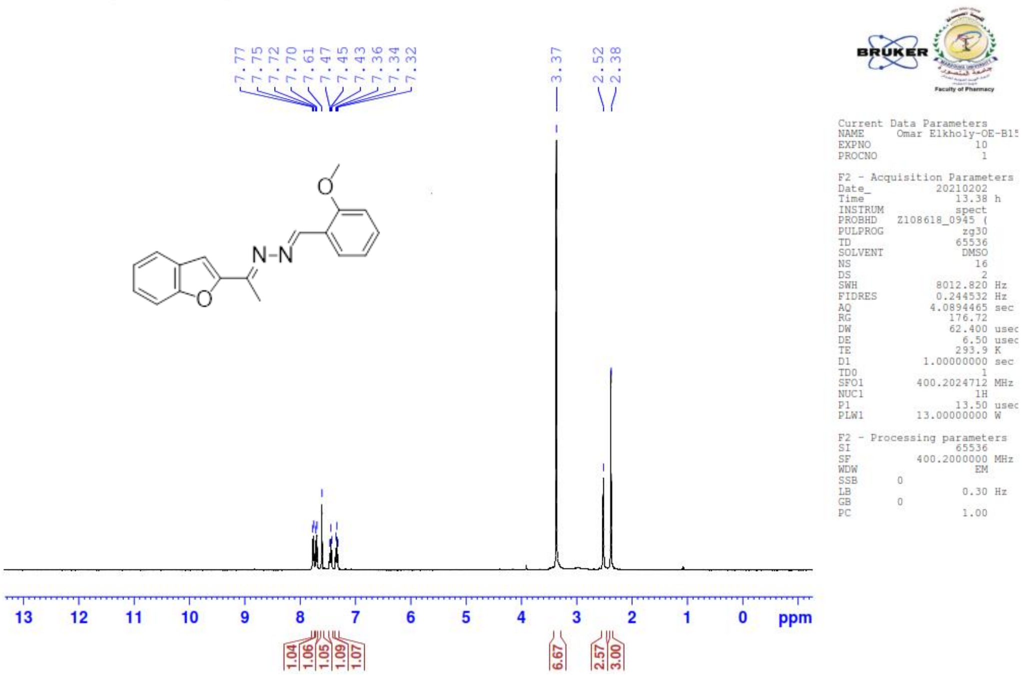

^{13}CNMR

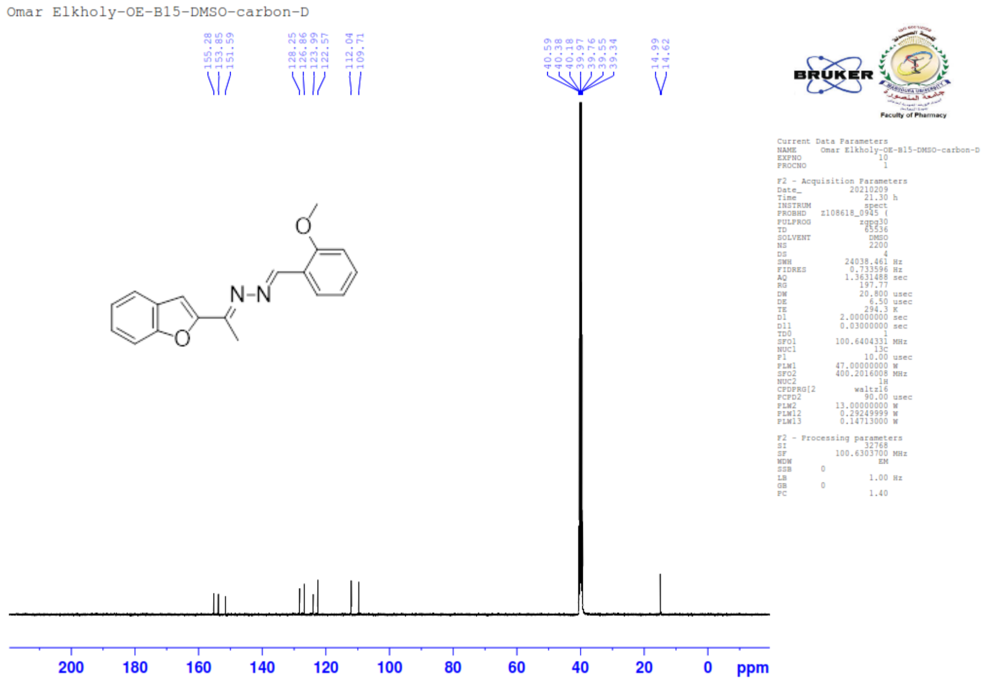

(E)-1-[1-(benzofuran-2-yl)ethylidene]-2-((E)-4-hydroxybenzylidene)hydrazine **(11)**

^{1}HNMR

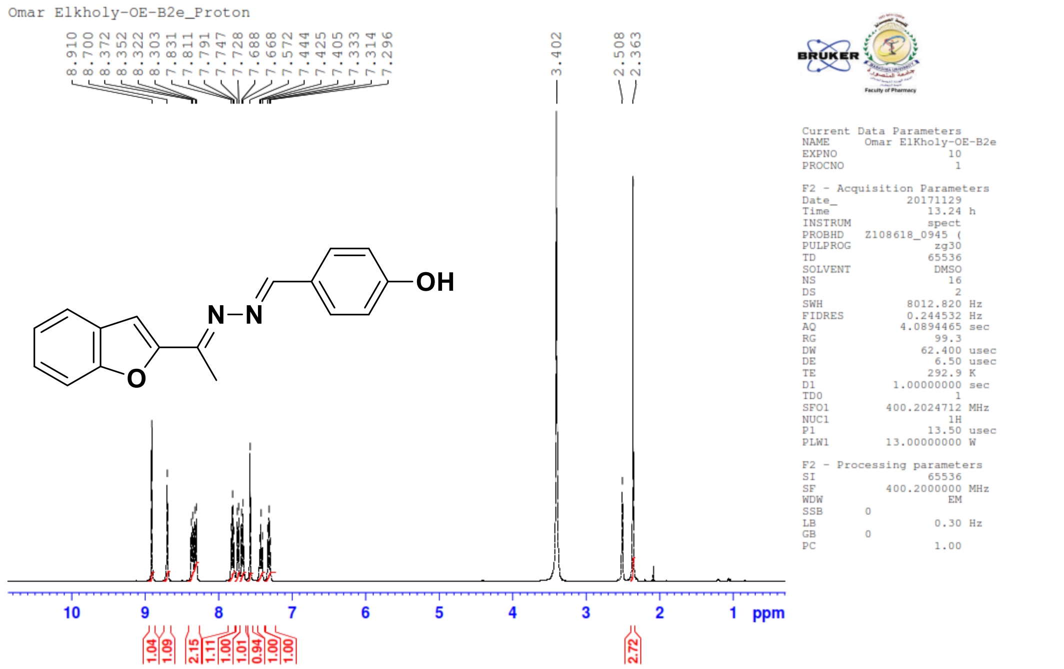

^{13}CNMR

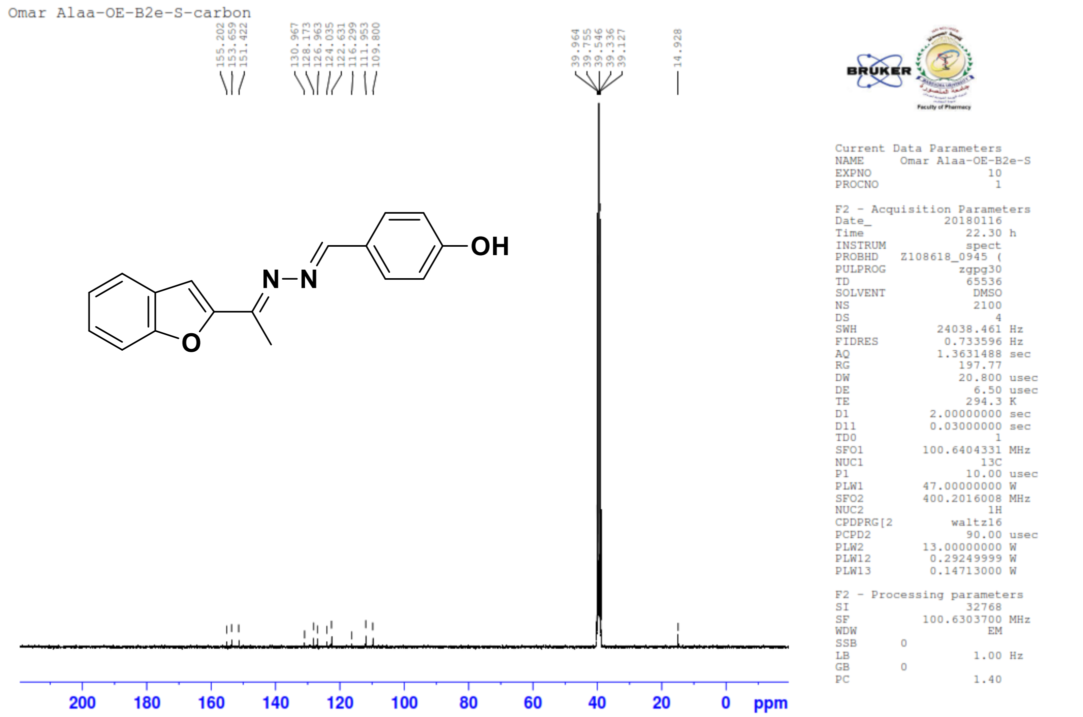

(1E,2E)-1-[1-benzofuran-2-yl) ethylidene]-2-((E)-3-phenylallylidene)hydrazine ***(12)***

^{1}HNMR

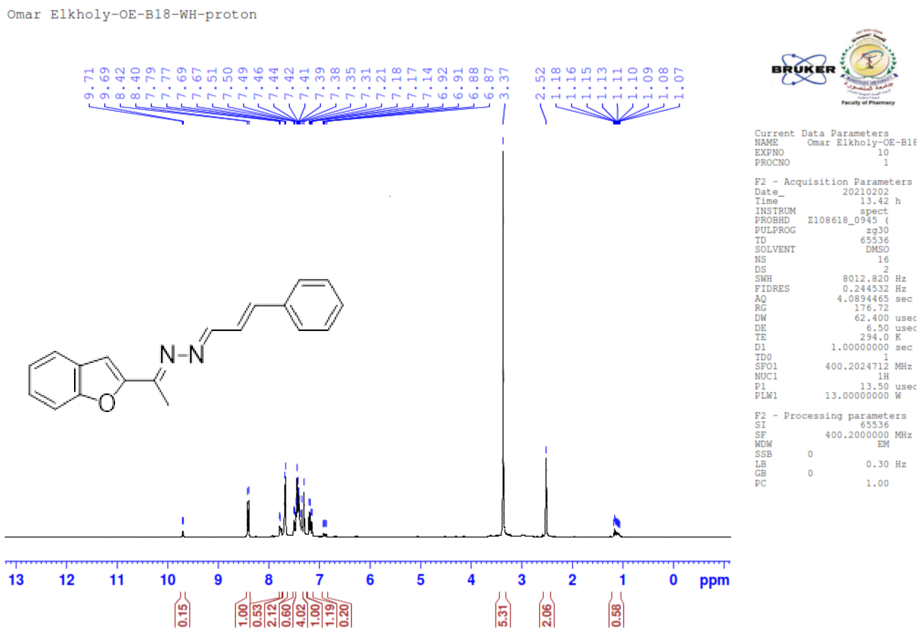

^{13}CNMR

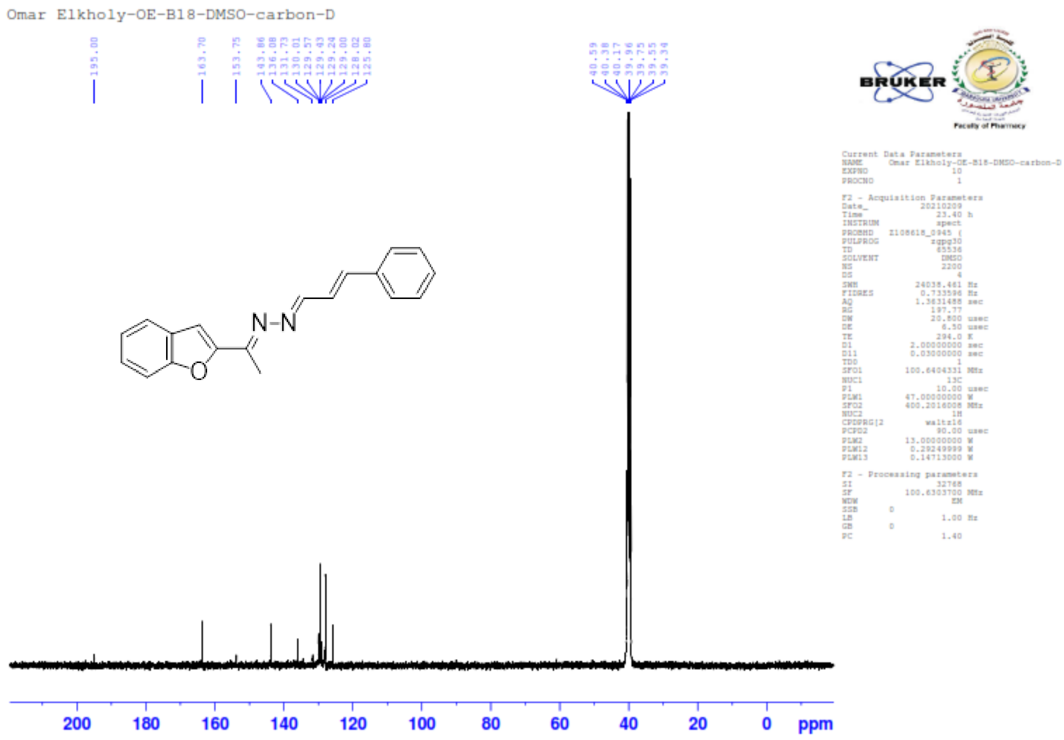

Cytotoxic activity of some compounds against human cell line

Comp. No.	Code.	In vitro Cytotoxicity IC50 (μM) •				
		WI38	HePG2	PC3	Hela	MCF7
••	DOX	6.72±0.5	4.50±0.2	8.87±0.6	5.57±0.4	4.17±0.2
7	OE-G1	>100	81.59±3.9	93.86±4.7	66.05±3.2	76.91±3.8
12	OE-B18	56.45±3.2	62.04±3.2	74.38±3.9	39.82±2.7	68.36±3.6
5	OE-J5	63.97±3.1	12.61±1.0	41.52±2.6	28.33±2.2	19.92±1.5
8	OE-G12	48.18±2.8	9.73±0.7	17.49±1.3	7.94±0.5	11.58±0.9
9	OE-B12	82.31±4.3	26.40±2.1	32.10±2.2	17.02±1.4	28.10±2.3
3	OE-E4	78.52±3.8	34.82±2.5	65.01±3.2	51.37±3.1	45.07±2.9
10	OE-B15	67.74±3.4	50.97±3.0	24.76±1.9	13.82±1.0	62.83±3.3
11	OE-B2e	43.25±2.9	84.92±3.2	>100	89.31±3.2	>100

• IC50 (μM) : 1 – 10 (very strong). 11 – 20 (strong). 21 – 50 (moderate). 51 – 100 (weak) and above 100 (non-cytotoxic)

•• DOX : Doxorubicin

Average of Relative viability of cells (%)

Conc.(µM)	WI38	HePG2	PC3	Hela	MCF7
**OE-G1** **Comp.7**					
100	55.5	48.1	50.9	42.9	47.8
50	76.2	60.2	64.8	56.3	58.0
25	89.3	72.5	76.5	68.4	70.2
12.5	100	87.4	93.6	80.7	88.9
6.25	100	99.7	100	93.8	97.6
3.125	100	100	100	100	100
1.56	100	100	100	100	100
**OE-B18** **Comp.12**					
100	41.0	39.4	47.3	34.1	43.2
50	52.5	54.0	55.0	41.8	57.1
25	61.9	70.5	71.7	59.2	70.2
12.5	78.1	91.3	86.6	72.3	81.8
6.25	91.3	100	98.4	86.6	94.7
3.125	100	100	100	100	100
1.56	100	100	100	100	100
**OE-J5** **Comp.5**					
100	38.2	15.3	32.9	26.3	20.4
50	57.1	24.0	47.8	38.1	32.5
25	72.3	37.2	58.1	51.4	41.8
12.5	91.8	42.3	67.2	62.9	54.9
6.25	100	65.0	92.7	79.3	72.1
3.125	100	76.8	100	96.6	96.2
1.56	100	100	100	100	100
**OE-G12** **Comp.8**					
100	36.4	13.2	19.3	8.2	13.7
50	48.2	20.8	27.1	16.0	21.2
25	62.7	29.1	39.2	24.5	28.3
12.5	75.6	24.9	51.6	32.8	42.9
6.25	89.3	67.3	72.9	53.7	65.7

**3.125**	**100**	**81.4**	**93.7**	**76.1**	**79.8**
**1.56**	**100**	**99.5**	**100**	**88.6**	**99.1**
**OE-B12**<br><br>**Comp.9**					
**100**	**47.9**	**26.2**	**31.2**	**20.3**	**25.3**
**50**	**60.6**	**36.8**	**44.8**	**28.2**	**38.2**
**25**	**74.5**	**49.6**	**52.6**	**37.1**	**50.6**
**12.5**	**85.2**	**60.5**	**60.2**	**50.4**	**64.2**
**6.25**	**99.7**	**78.2**	**75.8**	**73.2**	**79.5**
**3.125**	**100**	**94.4**	**96.1**	**92.0**	**96.0**
**1.56**	**100**	**100**	**100**	**100**	**100**
**OE-E4**<br><br>**Comp.3**					
**100**	**47.4**	**29.5**	**37.5**	**36.1**	**33.9**
**50**	**59.2**	**42.8**	**59.5**	**50.7**	**47.6**
**25**	**71.8**	**50.2**	**71.9**	**64.8**	**61.3**
**12.5**	**85.3**	**76.1**	**92.8**	**75.9**	**72.4**
**6.25**	**97.0**	**82.4**	**100**	**97.1**	**94.7**
**3.125**	**100**	**100**	**100**	**100**	**100**
**1.56**	**100**	**100**	**100**	**100**	**100**
**OE-B15**<br><br>**Comp.10**					
**100**	**43.3**	**38.1**	**23.5**	**17.0**	**40.4**
**50**	**54.8**	**50.2**	**37.0**	**25.8**	**58.0**
**25**	**71.7**	**63.1**	**46.8**	**33.7**	**65.2**
**12.5**	**93.6**	**72.7**	**59.1**	**48.1**	**79.3**
**6.25**	**100**	**91.9**	**78.2**	**63.5**	**94.7**
**3.125**	**100**	**100**	**96.3**	**85.9**	**100**
**1.56**	**100**	**100**	**100**	**100**	**100**

Cytotoxic activity of some compounds against human cell line

Comp. No.	Code.	In vitro Cytotoxicity IC50 (μM) •				
		WI38	HePG2	PC3	Hela	MCF7
••	DOX	6.72±0.5	4.50±0.2	8.87±0.6	5.57±0.4	4.17±0.2
7	OE-G1	>100	81.59±3.9	93.86±4.7	66.05±3.2	76.91±3.8
12	OE-B18	56.45±3.2	62.04±3.2	74.38±3.9	39.82±2.7	68.36±3.6
5	OE-J5	63.97±3.1	12.61±1.0	41.52±2.6	28.33±2.2	19.92±1.5
8	OE-G12	48.18±2.8	9.73±0.7	17.49±1.3	7.94±0.5	11.58±0.9
9	OE-B12	82.31±4.3	26.40±2.1	32.10±2.2	17.02±1.4	28.10±2.3
3	OE-E4	78.52±3.8	34.82±2.5	65.01±3.2	51.37±3.1	45.07±2.9
10	OE-B15	67.74±3.4	50.97±3.0	24.76±1.9	13.82±1.0	62.83±3.3
11	OE-B2e	43.25±2.9	84.92±3.2	>100	89.31±3.2	>100

• **IC50 (μM)** : 1 – 10 (very strong). 11 – 20 (strong). 21 – 50 (moderate). 51 – 100 (weak) and above 100 (non-cytotoxic)

•• **DOX** : Doxorubicin

Average of Relative viability of cells (%)

Conc.(µM)	WI38	HePG2	PC3	Hela	MCF7
**OE-G1**<br><br>**Comp.7**					
100	55.5	48.1	50.9	42.9	47.8
50	76.2	60.2	64.8	56.3	58.0
25	89.3	72.5	76.5	68.4	70.2
12.5	100	87.4	93.6	80.7	88.9
6.25	100	99.7	100	93.8	97.6
3.125	100	100	100	100	100
1.56	100	100	100	100	100
**OE-B18**<br><br>**Comp.12**					
100	41.0	39.4	47.3	34.1	43.2
50	52.5	54.0	55.0	41.8	57.1
25	61.9	70.5	71.7	59.2	70.2
12.5	78.1	91.3	86.6	72.3	81.8
6.25	91.3	100	98.4	86.6	94.7
3.125	100	100	100	100	100
1.56	100	100	100	100	100
**OE-J5**<br><br>**Comp.5**					
100	38.2	15.3	32.9	26.3	20.4
50	57.1	24.0	47.8	38.1	32.5
25	72.3	37.2	58.1	51.4	41.8
12.5	91.8	42.3	67.2	62.9	54.9
6.25	100	65.0	92.7	79.3	72.1
3.125	100	76.8	100	96.6	96.2
1.56	100	100	100	100	100
**OE-G12**<br><br>**Comp.8**					
100	36.4	13.2	19.3	8.2	13.7
50	48.2	20.8	27.1	16.0	21.2
25	62.7	29.1	39.2	24.5	28.3
12.5	75.6	24.9	51.6	32.8	42.9
6.25	89.3	67.3	72.9	53.7	65.7

3.125	100	81.4	93.7	76.1	79.8
1.56	100	99.5	100	88.6	99.1
OE-B12<br><br>Comp.9					
100	47.9	26.2	31.2	20.3	25.3
50	60.6	36.8	44.8	28.2	38.2
25	74.5	49.6	52.6	37.1	50.6
12.5	85.2	60.5	60.2	50.4	64.2
6.25	99.7	78.2	75.8	73.2	79.5
3.125	100	94.4	96.1	92.0	96.0
1.56	100	100	100	100	100
OE-E4<br><br>Comp.3					
100	47.4	29.5	37.5	36.1	33.9
50	59.2	42.8	59.5	50.7	47.6
25	71.8	50.2	71.9	64.8	61.3
12.5	85.3	76.1	92.8	75.9	72.4
6.25	97.0	82.4	100	97.1	94.7
3.125	100	100	100	100	100
1.56	100	100	100	100	100
OE-B15<br><br>Comp.10					
100	43.3	38.1	23.5	17.0	40.4
50	54.8	50.2	37.0	25.8	58.0
25	71.7	63.1	46.8	33.7	65.2
12.5	93.6	72.7	59.1	48.1	79.3
6.25	100	91.9	78.2	63.5	94.7
3.125	100	100	96.3	85.9	100
1.56	100	100	100	100	100

Researcher : Dr.Omar elkholy email: dr.kholy93@gmail.com mob. 01278084495
Date : 23-03-2021
Assay : PI3K-α assay
Samples : 01 sample .
Reference : ---
Cell lines : ---
Kit used : ---
Solvent : DMSO
Assay samples : ---

Lab Report

**ser**	**compound**			PI3K-α **IC50** **ng/ml**	SD. ±
	code	**M.W**	**conc**		
1	OE-G12			2.21	0.11
***	LY294002			8.04	0.42

Detailed results:

code	IC50	conc	log	%inh	T2	T1	ΔT	RFU2	RFU1	ΔRFU	slope	K.Activity	EC
PI3K-α	[redacted]												
code	IC50	conc	log	%inh	T2	T1	ΔT	RFU2	RFU1	ΔRFU	slope	K.Activity	EC
OE-G12		100	2	80.7	30	0	30	19.26	0	19.26	3.33333	23.112023	120
		10	1	67	30	0	30	32.98	0	32.98	3.33333	39.57604	120
		1	0	42.7	30	0	30	57.31	0	57.31	3.33333	68.772069	120
		0.1	-1	22.1	30	0	30	77.91	0	77.91	3.33333	93.492093	120
EC				0	30	0	30	100	0	100	3.33333	120	120
	[redacted]												
code	IC50	conc	log	%inh	T2	T1	ΔT	RFU2	RFU1	ΔRFU	slope	K.Activity	EC
LY294002		100	2	76.7	30	0	30	23.26	0	23.26	3.33333	27.912028	120
		10	1	54.8	30	0	30	45.18	0	45.18	3.33333	54.216054	120
		1	0	21.2	30	0	30	78.76	0	78.76	3.33333	94.512095	120
		0.1	-1	8.66	30	0	30	91.34	0	91.34	3.33333	109.60811	120
EC				0	30	0	30	100	0	100	3.33333	120	120
			[redacted]										

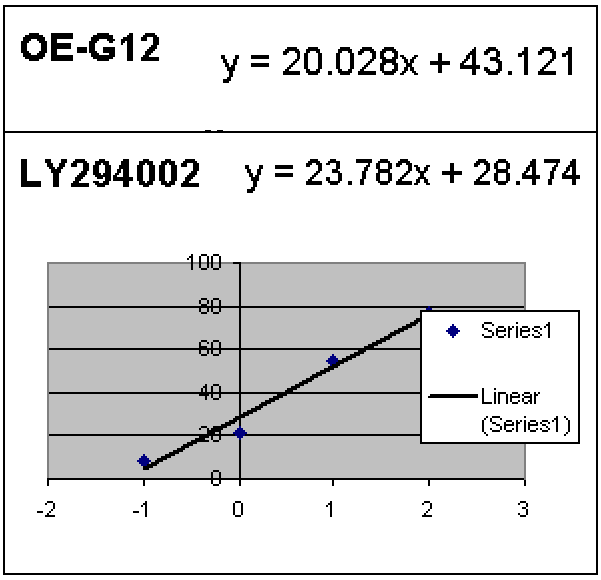

Researcher : Dr.Omar elkholy email: dr.kholy93@gmail.com mob. 01278084495
Date : 23-03-2021
Assay : VEGFR2 assay
Samples : 01 sample .
Reference : ---
Cell lines : ---
Kit used : ---
Solvent : DMSO
Assay samples : ---

Lab Report

**ser**	**compound**			**VEGFR2**	SD. ±
	code	**M.W**	**conc**	**IC50 ng/ml**	
1	OE-G12			86	4
***	Sorafenib			34	1

<u>Detailed results:</u>

vegfr2	[redacted]												
code	IC50	conc	log	%inh	T2	T1	ΔT	RFU2	RFU1	ΔRFU	slope	K.Activity	EC
OE-G12		10	1	90.4	30	0	30	9.59	0	9.59	3.33333	11.508012	120
		1	0	75.3	30	0	30	24.71	0	24.71	3.33333	29.65203	120
		0.1	-1	52.7	30	0	30	47.26	0	47.26	3.33333	56.712057	120
		0.01	-2	28.7	30	0	30	71.33	0	71.33	3.33333	85.596086	120
EC				0	30	0	30	100	0	100	3.33333	120	120
	[redacted]												
code	IC50	conc	log	%inh	T2	T1	ΔT	RFU2	RFU1	ΔRFU	slope	K.Activity	EC
sorafenib		10	1	93.1	30	0	30	6.88	0	6.88	3.33333	8.2560083	120
		1	0	81.4	30	0	30	18.64	0	18.64	3.33333	22.368022	120
		0.1	-1	61.7	30	0	30	38.25	0	38.25	3.33333	45.900046	120
		0.01	-2	36.8	30	0	30	63.23	0	63.23	3.33333	75.876076	120
EC				0	30	0	30	100	0	100	3.33333	120	120
			[redacted]										

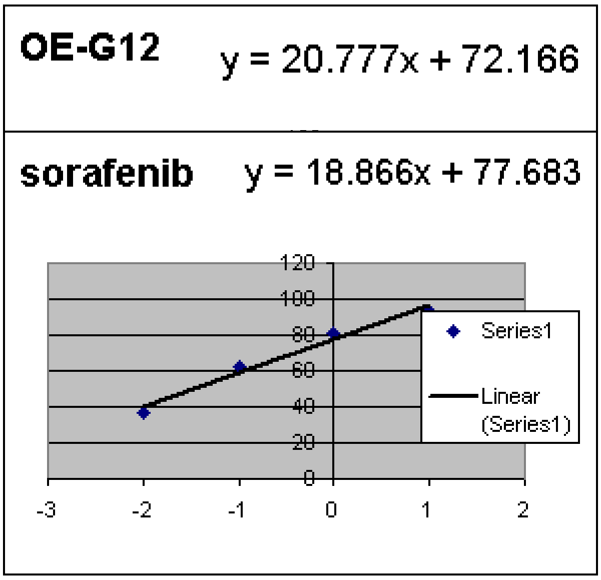

Researcher : Dr.Omar El-Khouly email : dr.kholy93@gmail.com mob. 01278084495
Assay : Cell Cycle Analysis
Samples : 02 samples
cell line : ---
Ref. : ---
Date : 23-03-2021
Reader : BD FACSCalibur
Kit used : ab139418_Propidium Iodide Flow Cytometry Kit/BD
Solvent : DMSO
Assay samples : Cell culture

Lab Report

**Ser**	Sample data		Results DNA content				
	**code**	**IC50 uM**	%G0-G1	%S	%G2/M	%Pre-G1	Comment
1	OE-G12/Hela		47.06	51.23	1.71	24.71	cell growth arrest@G1/S phase
2	Cont. Hela		46.26	42.99	10.75	1.95	---

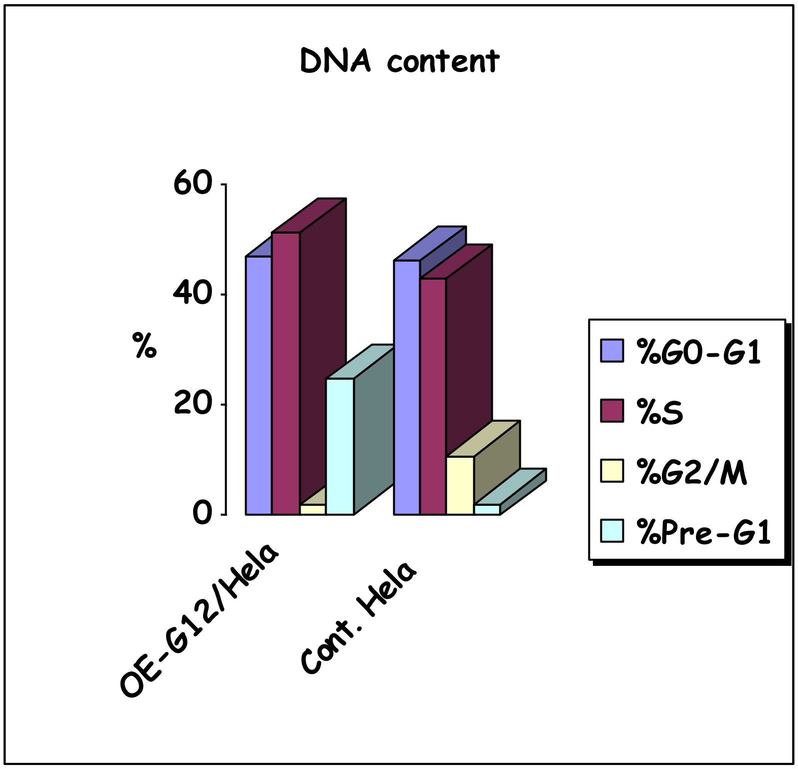

s	code	conc	Apoptosis			Necrosis
			Total	Early	Late	
1	OE-G12/Hela		24.71	1.79	14.11	8.81
2	Cont. Hela		1.95	0.63	0.17	1.15

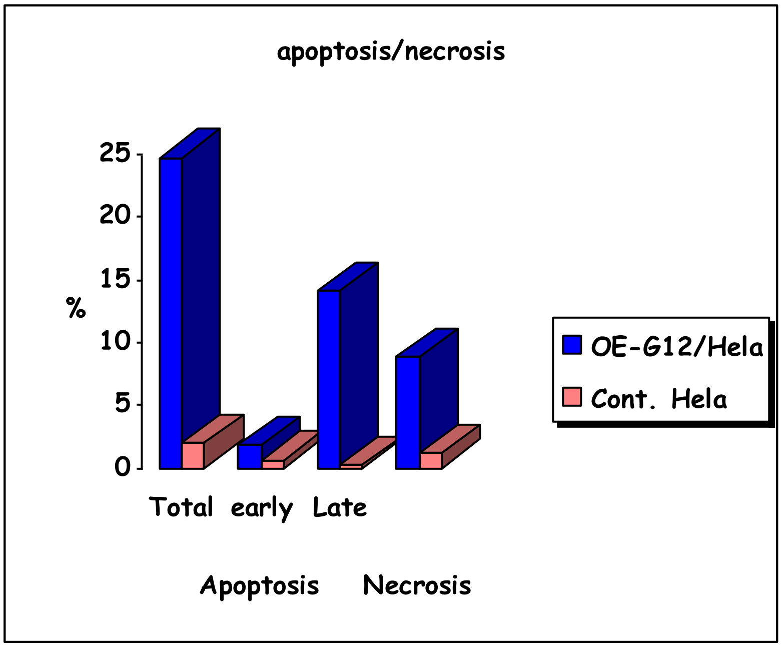

Detailed results

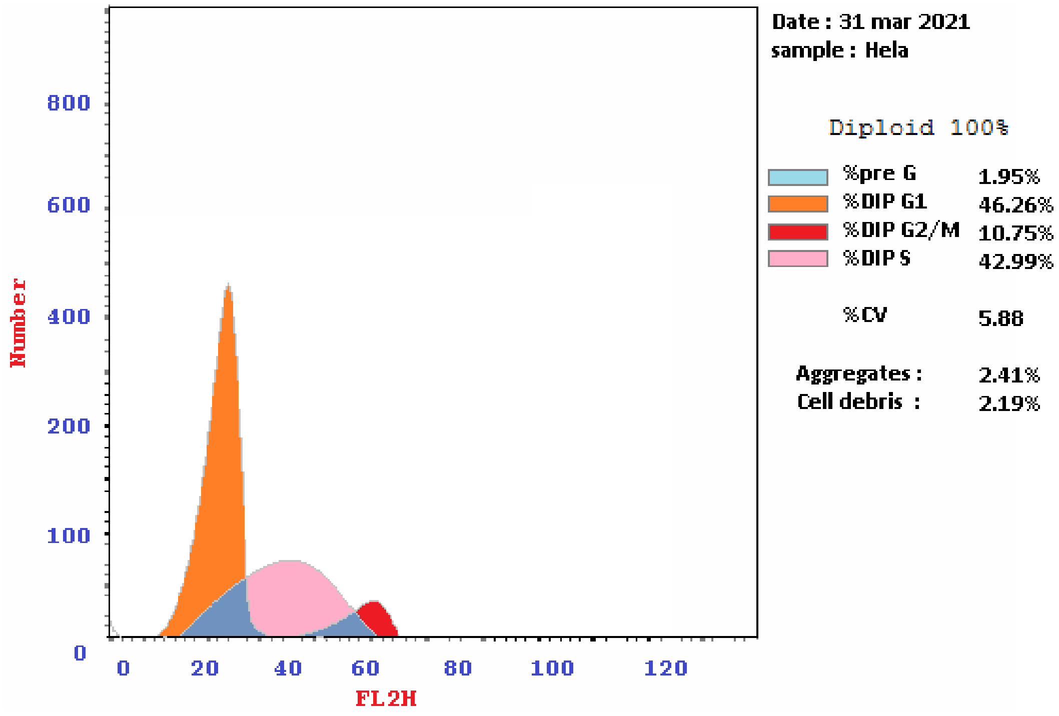

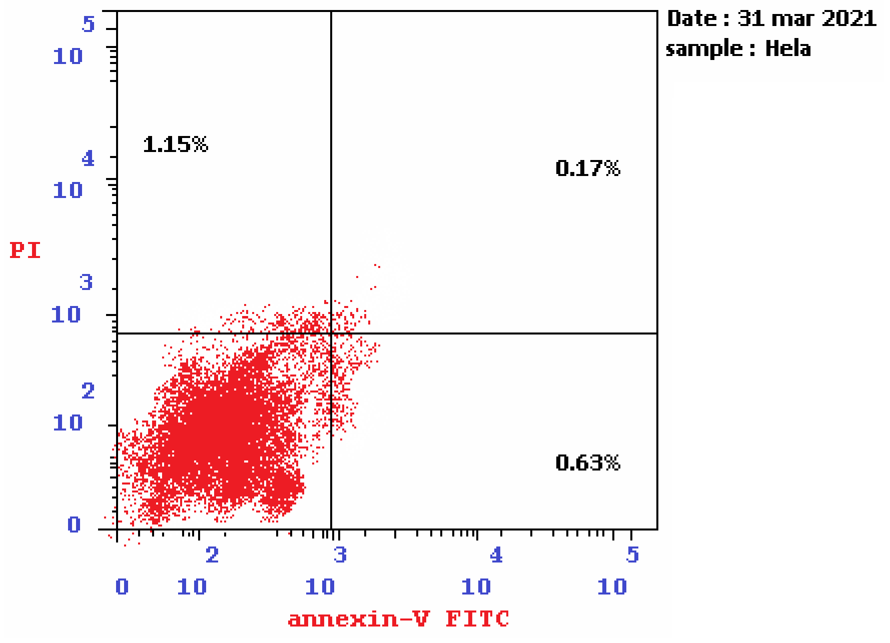

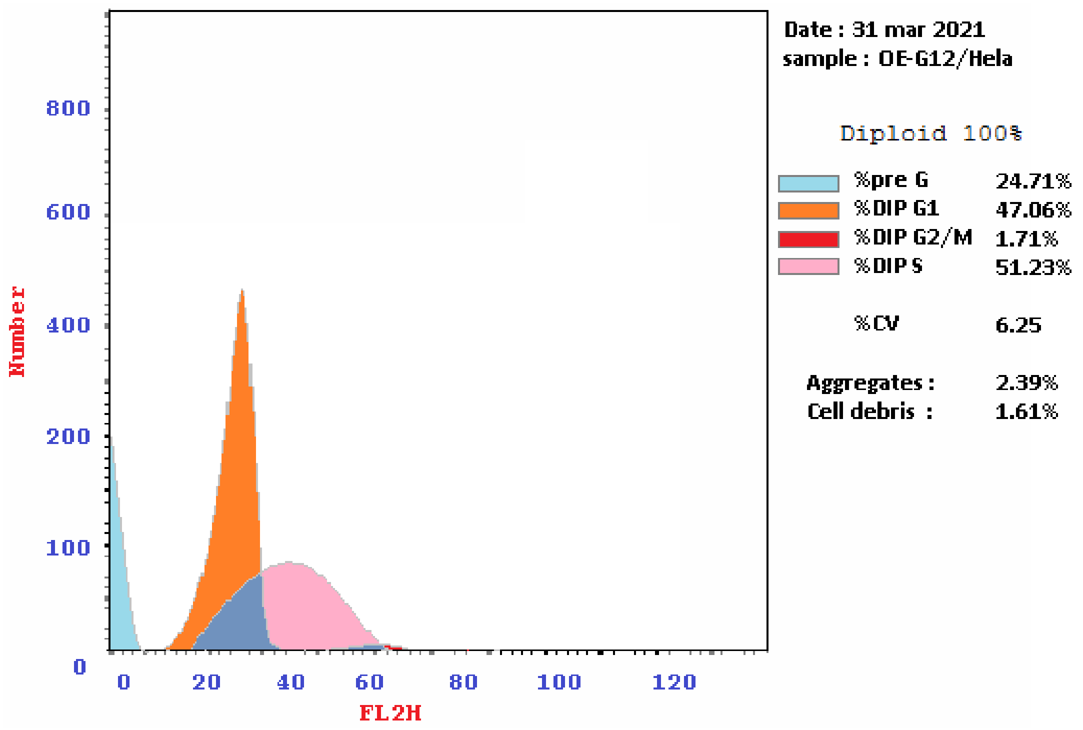
Date : 31 mar 2021
sample : OE-G12/Hela
Diploid 100%
%pre G 24.71%
%DIP G1 47.06%
%DIP G2/M 1.71%
%DIP S 51.23%
%CV 6.25
Aggregates : 2.39%
Cell debris : 1.61%
Number
800
600
400
200
100
0
0
20
40
60
80
100
120
FL2H

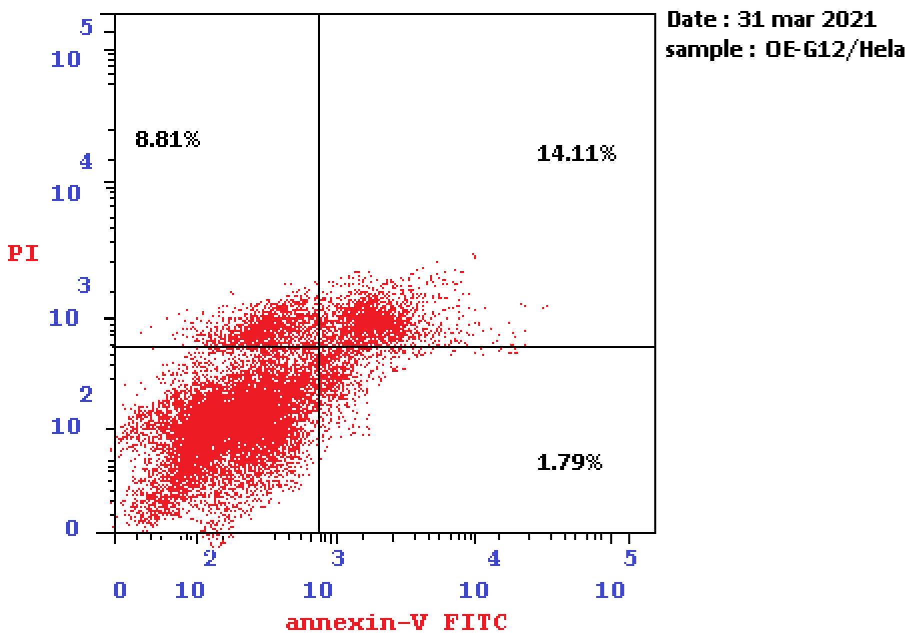
Date : 31 mar 2021
sample : OE-G12/Hela
8.81%
14.11%
1.79%
PI
annexin-V FITC
10^5
10^4
10^3
10^2
0
0
10^2
10^3
10^4
10^5
